# Court‐mandated interventions for individuals convicted of domestic violence: An updated Campbell systematic review

**DOI:** 10.1002/cl2.1151

**Published:** 2021-03-14

**Authors:** David B. Wilson, Lynette Feder, Ajima Olaghere

**Affiliations:** ^1^ George Mason University Fairfax Virginia USA; ^2^ University of Central Florida Orlando Florida USA; ^3^ Temple University Philadelphia Pennsylvania USA

## Abstract

**Background:**

Survey research and analysis of police records, hospital emergency rooms, and women's shelters have clearly established the severity of the intimate partner violence problem and the need to find programs to address this issue. Roughly 1 in 4 women in an intimate relationship is a victim of intimate partner violence. Court‐mandated batterer intervention programs (BIPs) have been implemented throughout the United States as a leading method to address this problem. These programs are also now implemented in Canada and Europe. These programs emerged from the women's shelter movement leading to programs with a strong feminist orientation, such as the Duluth Model. The programs that were developed were group‐based and relied on psychoeducational methods. Their aim was to get men to take responsibility for their sexist beliefs and stop abusing their partners by teaching them alternative responses for handling their anger. More recent programs draw from cognitive‐behavioral therapeutic principles or a mix of the latter with feminist components as well.

**Objectives:**

This is an update of our prior review. The aim was to assess the effects of postarrest court‐mandated interventions for intimate partner violence offenders that target, in part or exclusively, male batterers. Our focus was on studies aimed at reducing intimate partner violence, above and beyond what would have been expected by routine legal procedures (e.g., probation monitoring, etc.).

**Search Methods:**

We searched numerous databases and websites, bibliographies of published reviews of related literature, and a scrutiny of annotated bibliographies of related literature. Our goal was to identify all published and unpublished literature that met our selection criteria. The original review identified nine eligible studies. The updated search identified two new studies. The total sample size across these 11 studies was 4824.

**Selection Criteria:**

We included experimental (random assignment) and quasi‐experimental evaluations of court‐mandated BIPs that measured official or victim reports of future intimate partner violence. Rigorous quasi‐experimental designs were defined as those that either used matching or statistical controls to improve the comparability of the treated (program) and untreated (comparison) groups. The original review also included quasi‐experimental designs that used treatment drop‐outs as the comparison group. Given the serious selection bias of such studies, these have not been included in this update.

**Data Collection and Analysis:**

We coded characteristics of the treatment, sample, outcomes, and research methods. Findings were extracted in the form of an effect size and effect sizes were analyzed using the inverse‐variance weight method of meta‐analysis. Official report and victim report outcomes were analyzed separately as were the different design types (i.e., random assignment and quasi‐experimental designs with a no treatment comparison).

**Results:**

The mean effect for official reports of intimate partner violence from experimental studies showed a modest (but statistically nonsignificant) benefit for the program group (odds ratio, 0.79; 95% confidence interval [CI] [0.49–1.28], *k* = 7) whereas the mean effect for victim reported outcomes showed equal outcomes for both groups (e.g., no benefit or harm; odds ratio, 0.99; 95% CI, [0.74–1.32], *k* = 7). The quasi‐experimental studies showed a small but not statistically significant benefit for the program group on official reports (odds ratio, 0.54; 95% CI [0.24–1.22], *k* = 7). One quasi‐experiment reported a nonsignificant effect for a victim report outcome (odds ratio, 1.76; 95% CI [0.50–6.14], *k* = 1). None of the analyses were statistically significant. Thus, there is insufficient evidence to conclude that these programs are effective. Both the official measure and the victim reported measures have potential sources of bias, increasing the uncertainty regarding any benefits or harms related to these programs.

**Authors' Conclusions:**

The findings, we believe, raise doubts about the effectiveness of court‐mandated BIPs in reducing re‐assault among men convicted of misdemeanor intimate partner violence. New programs and/or entirely new approaches to this important social problem should be explored.

## 1 PLAIN LANGUAGE SUMMARY

Insufficient evidence to determine the effectiveness of court‐mandated interventions for men convicted of domestic violence.

Intimate partner violence affects roughly 1 in 4 women in an intimate relationship, although estimates vary by country. Intimate partner violence also accounts for roughly 11% of all homicides in the United States. Men are also victims of intimate partner violence, but male victims are not the focus of this review.

One approach to addressing this problem common in the United States is a court‐mandated group‐based batterer intervention program (BIP) for male batterers.

### 1.1 What is this review about?

The objective of this review was to determine if court‐mandated group‐based BIPs are effective at reducing intimate partner violence among male batterers.

**What is the aim of this review?**

This update of a Campbell systematic review examines the effects of court‐mandated batterer intervention programs for adult males who have perpetrated intimate‐partner violence.

### 1.2 What studies are included?

The review summarizes the evidence from 11 high‐quality studies, including four randomized controlled trials and eight quasi‐experimental comparison group studies.

Eight studies were conducted in the United States, two in Canada and one in Australia.

### 1.3 What are the main findings of this review?

The studies we included do not support the effectiveness of court‐mandated BIPs. There are two important caveats.

First, there is not enough evidence to draw a strong conclusion that these programs do not work. The evidence is insufficient to conclude that they do work.

Second, there is a new generation of these programs that have incorporated new elements, such as motivational interviewing. Meta‐analyses have established that motivational interviewing improves in‐program outcomes, such as attendance and other indicators of compliance, but there is insufficient evidence to establish whether these newer generation programs reduce postprogram intimate partner violence.

### 1.4 What do the findings of this review mean?

The classic BIP that relied solely on a feminist framework, a cognitive‐behavioral model, or a mix of the two, is unlikely to provide a meaningful solution to the problem of intimate partner violence. New programs and/or entirely new approaches to this important social problem should be explored.

### 1.5 How up‐to‐date is this review?

This authors of this review update searched for studies up to February 2018.

## 2 BACKGROUND

### 2.1 The problem, condition, or issue

The Centers for Disease Control and Prevention (CDC) defines intimate partner violence as, “physical violence, sexual violence, threats of physical/sexual violence, and psychological/emotional abuse perpetrated by a current or former spouse, common‐law spouse, nonmarital dating partners, or boyfriend/girlfriends of the same or opposite sex” (Saltzman et al., 1999). Research indicates how pervasive the problem of intimate partner violence is today. A systematic review by Desmarais et al. (2012) indicated that over 20% of women in a heterosexual intimate relationship experienced physical violence. Examining data from 81 countries, Krahé (2018) estimated that roughly 30% of all women over the age of 15 who have been in an intimate partner relationship at some point experience physical and/or sexual assault (see also Desmarais et al., 2012). Krahé (2018) further discusses the increased risk of negative mental and physical health outcomes for women who are victims of intimate partner violence.

Within the United States, intimate partners committed 14% of all homicides with females making up the vast majority of victims killed by an intimate partner (Catalano et al., 2009). The National Crime Victimization Survey indicated that there were 847,230 violent crimes committed against persons by their current or former spouses or significant others in 2018 in the United States (Morgan & Oudekerk, 2019). Based on the CDC's National Intimate Partner and Sexual Violence Survey, researchers estimated that one in four women experience an intimate partner violent episode sometime during their lifetime (Smith et al., 2018). Beyond the harm to these victims, a nationally representative sample calculated that approximately 7 million children live in families with severe partner violence (McDonald et al., 2006) thereby increasing the likelihood of continuing the cycle of violence. These numbers speak to the importance of finding programs that can successfully intervene with intimate partner violence offenders.

An initial body of individual studies evaluating court‐mandated BIPs provided mixed findings on their effectiveness (Babcock et al., 2004; Davis & Taylor, 1999). While this first wave of evaluation research consistently indicated high rates of success, their findings likely reflected the methodological shortcomings of the research rather than the programs' actual effectiveness in reducing intimate partner violence. For example, several of these studies used program dropouts as the comparison group, likely producing overestimates of any benefits of these programs. That is, individuals who drop out of treatment might not be as motivated to change as those who complete these programs. These initial promising, but flawed, studies were followed by a period in which more rigorous research was conducted. Unlike the earlier research, these studies produced mixed results regarding the effectiveness of mandated BIPs in reducing intimate partner violence. These mixed results possibly reflected differences in the rigor of the research methodology used to evaluate these programs along with differences in outcome measures utilized, length of time followed, and the integrity with which the intervention was implemented apart from additional programs and services that may have been provided at the different intervention sites.

### 2.2 Prior reviews

To date, two meta‐analyses other than the prior version of this review have been conducted studying the effectiveness of court‐mandated counseling in reducing future violence among intimate partner violence offenders. Davis and Taylor (1999) included five quasi‐experimental studies using a nonequivalent matched group design (they discarded one study because its results were viewed as anomalous) and two experimental studies with random assignment. They concluded that, “among the handful of quasi‐ and true experiments there is fairly consistent evidence that treatment works and that the effect of treatment is substantial” (Davis & Taylor, 1999, p. 69). Their analysis found a fairly substantial mean effect size (*d*) of 0.412 for experimental studies and 0.416 for quasi‐experimental studies.

Babcock et al. (2004) examined a larger number of evaluations in their systematic review and meta‐analysis. Their search yielded 17 quasi‐experimental studies (where treatment completers were compared to treatment dropouts, no‐shows and/or treatment rejects or to a matched comparison group that did not receive treatment) and 5 experimental designs (with random assignment to treatment and control conditions). Babcock et al. (2004) found a small effect, which translated to a woman being “5% less likely to be assaulted by a man who was arrested, sanctioned, and went to a batterers' program than by a man who was simply arrested and sanctioned” (p. 1004). However, their inclusion of all quasi‐experimental studies, including those failing to establish preintervention equivalency, in addition to not separately analyzing effect sizes for these different types of quasi‐experimental studies, may have created a bias in favor of finding positive results. This is an important consideration as prior research indicates research design can influence the likelihood of finding treatment effectiveness (see Feder & Forde, 2000; Weisburd et al., 2001).

More recent narrative and vote‐counting reviews have come to similar equivocal conclusions regarding the effectiveness of these programs. For example, Stover et al. (2009) concluded that “extant interventions have limited effect on repeat violence” (p. 223). A vote‐counting review by Eckhardt et al. (2013) concluded that the evidence is “very mixed” with regard to the effectiveness of BIP programs for perpetrators of intimate partner violence. Furthermore, there was little evidence to suggest one program type is more effective than another. This review was more inclusive than the current study in terms of program types (need not be court‐mandated) and identified 20 studies. The authors did, however, express some optimism for the effectiveness of some newer programs with alternative content. Finally, a recent review of BIPs in Spain by Ferrer‐Perez and Bosch‐Fiol (2018) concluded that the “methodological limitations of outcome studies still preclude our ability to determine the effectiveness of these interventions” (p. 891). It is worth noting that most of the studies identified by Ferrer‐Perez and Bosch‐Fiol examined changes in psychological variables of abusers presumed to be related to abusive behavior, rather than directly examining the actual behavior of repeat violence. In contrast, a rapid evidence review by Mazerolle et al. (2018) on criminal justice responses to domestic and family violence drew a more positive conclusion, stating that batterer programs are associated with reduced recidivism.

A recent meta‐analysis of seven studies comparing standard BIPs to these programs with an added preintervention motivational interviewing component showed that the addition of motivational interviewing improved intervention dose and reduced program drop‐outs but did not significantly reduce physical and psychological intimate partner violence (Santirso et al., 2020). Similarly, Soleymani et al. (2018) review of five studies supported that the addition of motivational interviewing increased the level of engagement, session attendance, and homework compliance.

The most recent reviews have been conducted by Arce et al. (2020) and Cheng et al. (2019). The Arce et al. (2020) review is an update of a prior review (Arias et al., 2013) and included 25 studies. There were a mix of studies with and without comparison groups. An effect size was conducted separately for each experimental and comparison/control group, and against a common assumed failure rate for each group across studies. These effect sizes were also upwardly adjusted (in absolute value) for measurement unreliability. Thus, even for those studies that were randomized control trials, the design was down‐graded to a single‐group study. We would argue that the findings from this review are uninterpretable. The Cheng et al. (2019) meta‐analysis included 14 studies and concluded that the randomized controlled trials failed to find evidence supporting the effectiveness of these programs whereas the quasi‐experimental studies collectively suggested some benefit. Similarly, a Cochrane systematic review of six randomized control trials of both voluntary and court mandated cognitive‐behavioral programs for male batterers found “no clear evidence of an effect” (p. 1).

Our systematic review and meta‐analysis, like the Babcock et al. (2004) review, tried to locate all studies conducted in the United States and elsewhere, whether or not it was published. Similarly, we included all experimental designs meeting our inclusion criteria. However, unlike the Babcock et al. review, we did not include all quasi‐experimental studies, but instead limited inclusion to those that addressed selection bias either via a matched group design or using statistical controls. This led to only including evaluations using more rigorous methods when comparing programs. Additionally, we provided separate analyses for each type of research design to decipher the effect of research design type on treatment effectiveness.

### 2.3 How the intervention might work

The idea of counseling male intimate partner violence offenders developed directly out of the women's shelter movement where advocates, working with battered women, realized that the only way to stop the cycle of violence was to change the behavior of the abuser (Feazell et al., 1984). It is not surprising, therefore, that these programs borrowed heavily from a feminist orientation. Typically, the various programs encouraged men to confront their sexist beliefs and accept responsibility for their past abuse, while teaching them alternative behaviors and reactions (e.g., anger management, assertiveness, relaxation techniques, and communication skills).

One of the earliest and more widely used programs is the Duluth model. Price and Rosenbaum (2009) found that 53% of 276 programs for perpetrators of intimate partner violence across 45 states within the United States used the Duluth model or a Duluth philosophy. The Duluth model is based on feminist and sociological frameworks, placing the causal mechanism for intimate partner violence at a societal level and rooted in patriarchal cultural norms (Babcock et al., 2016). The primary treatment method is psychoeducational and focuses on changing men's beliefs “about their privilege in society and the unequal, subservient position they believe women should maintain” (Miller, 2010; Pence et al. 1993 as cited in Babcock et al., 2016, p. 361). Thus, the presumed mechanism of change for the Duluth model and other programs that draw from this orientation is that cultivating a philosophy of gender parity among men will lead to a reduction or elimination of intimate partner violence (Babcock et al., 2016, p. 361).

In contrast to the feminist orientation of the Duluth model and related programs, many other programs draw from cognitive‐behavioral or other mental health frameworks. A survey of 238 North American programs by Cannon et al. (2016) found that roughly 30% used a cognitive‐behavioral model as the primary treatment approach and another 25% used this model as a secondary treatment approach. A survey of European programs by Hamilton et al. (2012) found that cognitive‐behavioral programs were the most widely used roughly 70% of the time.

Cognitive‐behavior programs assume that intimate partner violence is caused by “(i) cognitive distortions about self and partner and (ii) a lack of skills to appropriately express and process feelings leading to manipulative expressions of anger” (Banks et al., 2013, p. 161). Thus, these programs are presumed to work by addressing the distorted thinking and irrational thoughts among male participants. The programs typically include homework to help solidify the changes in cognitive processes. These approaches may also include behavioral components that address the deficits in skills related to dealing with anger.

An increasingly common component to cognitive‐behaviorally based programs is the addition of motivational interviewing or motivational planning as a preintervention adjunct (see, e.g., Alexander et al., 2010 and Santirso et al., 2020). The theory is that motivational interviewing will increase treatment compliance and engagement, thereby enhancing any treatment effect.

In practice, the survey of North American programs by Cannon et al. (2016) and the survey of European programs by Hamilton et al. (2012) showed that many programs blend the above approaches and may also include a mix of other techniques or philosophies. Thus, there may be multiple potential causal mechanism(s) for any given program and clearly differentiating between program types is difficult given their blended nature. This limited our ability to assess differential effectiveness by program type.

## 3 OBJECTIVES

In 1984, the Attorney General's Task Force on Family Violence recommended court‐mandated treatment as an addition to legal alternatives United States Attorney General's Task Force on Family Violence & United States, Department of Justice, Yet 35 years later, the field remains uncertain about whether these programs are more effective at reducing future intimate partner violence than legal interventions alone (e.g., arrest, prosecution, conviction and short jail stay and/or probation). The National Academy of Sciences has noted that “the urgency and magnitude of the problem of family violence have caused policy makers, service providers, and advocates to take action in the absence of scientific knowledge that could inform policy and practice” (Chalk & King, 1998, p. 2). Therefore, the aim of this systematic review and meta‐analysis is to assess the effects of postarrest court‐mandated interventions (including pretrial diversion programs) for intimate partner violence offenders in the United States and in other countries. Additionally, this review also assesses the effect of methodological design on outcome findings by investigating results by type of research design used as well as by outcome type. This work is an update of a prior Campbell review on this topic (Feder et al., 2008).

## 4 METHODS

### 4.1 Notes regarding update

This is an update of a prior Campbell review (Review: Feder et al., 2008; Protocol: Feder & Wilson, 2006). The updated search identified two new studies: Blatch et al. (2016) and Labriola et al. (2002). In the initial review, we included studies that compared program completers to program drop‐outs. The results for these studies were presented separately and the rationale for including these studies was to demonstrate how biased their findings were relative to more credible designs. These studies were commonly cited as evidence for the effectiveness of BIPs. These drop‐out designs do not, however, provide credible evidence of effectiveness as men who drop‐out of these programs are likely to be meaningfully different than men who complete the program (Shadish et al., 2002). As such, we have dropped them from the updated review. We also reclassified Palmer et al. (1992) as a quasi‐experimental design. In the original review it was listed as an experimental design based on the claim in the methods section that noted the use of a “block random procedure” (p. 278) to assign men to the treatment or control condition. However, the text clarifies that “subjects were assigned to treatment if a new group was to commence within 3 weeks; otherwise they became part of the control group” (p. 278). This is not a true random assignment procedure, but rather a quasi‐random one similar to what was used in Dutton (1986).

The other deviation from the original protocol was to add additional items related to risk‐of‐bias. These items are shown in Table 2.

### 4.2 Criteria for considering studies for this review

#### 4.2.1 Types of studies

Only studies using an experimental or rigorous quasi‐experimental design were included. Experimental designs were defined as those using random assignment to the treatment and control groups. Rigorous quasi‐experimental designs were defined as those addressing selection bias in the program and comparison groups through the use of multivariate statistical methods or a matched subject research design. For both experimental and quasi‐experimental designs, control conditions could be no‐treatment, or treatment as usual. That is, the no‐treatment control condition could include routine legal interventions such as probation or a short jail stay. We excluded, however, referral to counseling or alternative programs designed specifically to reduce intimate partner violence (beyond any deterrent effect of jail or probation). We also excluded quasi‐experimental designs that used treatment dropouts as the control condition.

#### 4.2.2 Types of interventions

The intervention involved a postarrest court‐mandated intervention that, in part or exclusively, was aimed at the batterer and had as its goal, decreasing the batterers' future likelihood of re‐assaulting that or other partners. As so defined, pretrial diversion programs were eligible for inclusion. The format could have included group, individual, or a combination of the two. Any batterer intervention model that meets these criteria was eligible.

#### 4.2.3 Types of participants

Only studies that used adult participants (defined as persons aged 18 years or older) experiencing intimate partner violence, in heterosexual relationships, whether presently or formerly married, separated, divorced, cohabiting, or dating were included in the meta‐analysis. The perpetrator of the violence must also have been male. Studies were included in the systematic review as long as these criteria were met, even if the study sample included others who fell outside these criteria, so long as the effect for male batterers could be determined.

#### 4.2.4 Types of outcome measures

In order for a study to have been included in this systematic review it had to use an outcome measure of repeat intimate partner violence obtained at least 6 months posttreatment. This was defined as 6 months from the time that the treatment ended, that is, the individual completed his court‐mandate. This criterion was based on Dunford's findings that evaluation studies collecting outcome data at the end of treatment were more likely to find effectiveness than those measuring outcomes for some period posttreatment (Dunford, 2000). This suggested that evaluations that were based solely on end‐of‐treatment assessments should be viewed cautiously. Additionally, to be included, a study had to include at least one outcome measure on repeat violence other than offenders' self‐reported repeat violence (although it may also include such measures). As such, studies that included victim reports of the offender's abusive behavior, such as the Conflict Tactics Scale (CTS), or official measures of recidivism including arrest, charges or convictions were eligible for inclusion.

It needs to be noted that studies which exclusively relied on attitudinal changes were not included in this meta‐analysis. Undoubtedly, any positive effects of these programs would be mediated by other changes, such as attitudes and the acquisition of anger management strategies. Changes in these intermediate outcomes would be encouraging and these changes might lead to benefits not detected in the outcomes examined. However, the primary purpose of these programs is a reduction in repeated partner abuse. Additionally, attitudinal changes would rely on batterers' self‐reports. Whether it is due to social desirability or to other unknown factors, more than a few researchers working in this field have found reason to doubt these accounts (Eckhardt & Utschig, 2007; Edleson & Brygger, 1995; Feder & Dugan, 2002; Follingstad & Rogers, 2013; Freeman et al., 2015; Helfritz et al., 2006; Tolman & Edleson, 1995). As such, our decision was to limit outcomes to measures of continued abuse of a partner.

#### 4.2.5 Effect size data

Finally, to be included, the study needed to have reported sufficient data to permit the computation of an effect size.

#### 4.2.6 Language

Only studies published in the English language were included because of the language limitations of the review team.

### 4.3 Search methods for identification of studies

Our goal was to identify and include all published and unpublished studies conducted in the United States or elsewhere from 1986 through January 2003, the initial search, that met our inclusion criteria. Toward this aim, the first author (Lynette Feder), who had worked in this field for many years, canvassed a number of other researchers for additional studies, published or not, on the effectiveness of BIPs. The research team also searched databases and websites, bibliographies of published reviews of related literature and a scrutiny of annotated bibliographies of related literature (see below). The list is grouped in terms of those focused on: (1) published materials; (2) nonpublished materials; (3) governmental publications; and (4) existing registries of studies on intimate partner violence. It must be noted that some of the databases could be listed in multiple groups. That is, contained under “Published Materials” is Sociological Abstracts, Educational Resources Information Clearinghouse (ERIC), Criminal Justice Abstracts and others that contain unpublished as well as published literature with some containing international as well as national studies. Searches were conducted using the following databases and websites:

(1) *Published materials*

- PsycINFO
- ERIC
- MEDLINE
- Sociological Abstracts
- Social Science Citation Index
- Lexis Nexis Legal
- Lexis Nexis Medical
- Social Work Abstracts
- Criminal Justice Abstracts

(2) *Nonpublished materials*

- Dissertation Abstracts International

(3) *Governmental*

- GPO Monthly Catalog (MOCAT)
- National Criminal Justice Research Service
- UK National Health Service NRR (National Research Register)

(4) *Existing registers or studies on intimate partner violence*

- Social, Psychological, Criminological and Educational Trials Register (C2‐SPECTR)^1^
- PsiTri database of randomized and controlled trials in mental health
- Babcock and Taillade (1999)
- Davis and Taylor (1999)
- Babcock et al. (2004).

#### 4.3.1 Terms used to search

We used 25 keywords in three clusters to search for all experimental and quasi‐experimental studies conducted on the effectiveness of court‐mandated interventions for intimate partner violence offenders. Whenever appropriate a “wildcard” was used to search for the root of the word allowing for other possible derivations. For instance, the term “eval*” was used to capture “evaluation,” “evaluate,” “evaluating,” and so forth. The first cluster of keywords related to the subject matter. Cluster two sought to find citations using program or evaluation research keywords. Finally, cluster three used keywords related to outcomes. Terms within a cluster were connected with the Boolean “OR” (i.e., an abstract with any one of the terms got selected) and the clusters were then connected with the Boolean “AND” (i.e., an abstract with at least one of the terms in each cluster got selected). The search query was restricted to searching for keywords within the titles and abstracts of references. The keywords within each cluster were:

##### Cluster one—Subject words

“Anger management” OR Batter*(er/s) OR “Domestic assault” OR “Domestic violence” OR “Family violence” OR “Spous*(e/al) abuse” OR “Physical abuse” OR “Minneapolis Model” OR Duluth OR “Intimate partner violence

##### Cluster two—Program words

Defer*(ral/ring/rred) OR Program(s) OR Treatment(s) OR Intervention(s) OR Diversion*(ary) OR Prosecu*(te/tion/torial

##### Cluster three—Outcome words

Effect*(s/ive/iveness) OR Research(es) OR Outcome(s) OR Eval*(uation/luations/ating) OR Experiment*(al) OR Quasi(‐experimental) OR Random(ly) OR Compar*(ison/ing) OR Match*(ed/es/ing)

#### 4.3.2 Initial 2003 search

The first author and a graduate research assistant reviewed the titles and abstracts of studies identified through the search process. Studies that appeared likely to be eligible were retrieved in their entirety. The first author and a graduate research assistant were also responsible for reviewing the full text of all studies retrieved in their entirety to determine final eligibility in the meta‐analysis. Where disagreements or uncertainties regarding the inclusion of a study arose, the second author's opinion was sought to resolve differences and reach consensus.

#### 4.3.3 Updated 2018 search

For the updated search, title and abstract screening and duplicate removal was completed by a research assistant. After executing the search and culling a potentially eligible pool of studies, titles and abstracts were screened and if any references looked promising, the entire study was pulled and reviewed. The full text of 124 eligible titles were then retrieved and evenly divided by study author last name for full‐text review. The first and second authors completed title and abstract screening to further determine inclusion in the meta‐analysis.

#### 4.3.4 Criteria for determination of independent findings

To avoid the “double counting” of findings, two issues need to be addressed. The first is multiple publications based on the same data or research study. In these cases, the multiple publications were treated as a single study. Coding was based on all available publcations because each publication may have provided unique and useful information. The second issue is multiple findings from a single study. These were categorized by outcome construct (i.e., official report and victim report) and only a single effect per construct was used in any analysis. For the official report effect sizes, a decision rule was established for determining which effect size to use in an analysis if multiple effects were available. Preference was given to measures of arrest over conviction, as arrests involve fewer decisions on the part of the criminal justice system than convictions. Preference was also given to estimates that adjusted for baseline features over nonadjusted estimates. Additionally, effect sizes reported for a longer time‐frame (e.g., 12‐months instead of 6‐months) were selected over those of a shorter time‐frame. The logic was to select an official report that was as close to the behavior of interest (intimate partner violence) as possible. For victim report measures, all effect sizes measuring intimate partner violence were averaged and the composite was used in the analyses. The *SE* for the composite effect size was also the average of the individual *SE*s. Because these effect sizes were Hedges' *g* with a common sample size, the average *SE* differed little from the minimum or maximum *SE* for the effect sizes averaged within a study. As with official reports, effect sizes with a longer time‐frame or follow‐up period were selected and averaged, excluding the same constructs measured at an earlier time point.

Finally, a sensitivity analysis was performed that used all coded effect sizes using the robust *SE* method for addressing dependence across effect sizes. The helped ensure that the results were not unduly influenced by the above selection rules.

### 4.4 Data collection and analysis

#### 4.4.1 Study coding

A coding protocol was developed to capture information about the treatment programs, participants, and research methods. In addition, all outcomes of interest were coded as an effect size along with related information. The coding protocol also allowed for the coding of multiple effect sizes per study. Coding was completed using paper forms that resembled a survey form. The data were entered into a computer datafile for analysis and storage. All studies were double‐coded with differences resolved through consensus meetings. For the original review, all coding was done by Lynette Feder and David Wilson. For the updated review, all coding was done by David Wilson and Ajima Olaghere. The coding forms are provided in Supporting Information Appendix A. Coding items were developed for each of the following areas:

(1) Treatment: type of treatment, participant dropout from treatment, treatment integrity, length of treatment, treatment setting, treatment provider, treatment philosophy.
(2) Participants: representativeness of sample, age, geographic location.
(3) Research methods: nature of the assignment to conditions, integrity of the assignment process, study level attrition, differential attrition between conditions, use of statistical controls, use of matching.
(4) Effect size: data necessary for computation of the effect size (sample sizes, proportions, frequencies, etc.), nature of the outcome measure, source of the outcome measure (victim reports and/or police records), time frame for the outcome measure.
(5) Risk‐of‐bias: Several items related to risk‐of‐bias were also coded. These addressed issues related to the research design, the sample sizes, how the data were analyzed, potential selection bias, attrition, and selective reporting of outcomes. See Supporting Information Appendix A and Table 2 for details.

#### 4.4.2 Data synthesis

This systematic review used standard inverse‐variance weighted meta‐analytic methods. Dichotomous program effects (e.g., re‐offend or not) were encoded as odds ratio effect sizes and continuous measures (e.g., victim‐reported abuse) were encoded as standardized mean difference effect sizes (Hedges' *g*). These latter measures were then converted to logged odds ratios for consistency of presentation by multiplying the logged odds ratios by 1.65 (the Cox's method of converting between *g* and the logged odds ratio). Effects representing unique constructs were analyzed separately (e.g., official report, victim report). All analyses used a random effects model estimating the random effects variance component via the method‐of‐moments method. These analyses were performed using the *metafor* package in R written by Wolfgang Viechtbauer (2010).

The analysis that used all coded effect sizes used the robust variance estimating method of Hedges et al. (2010) as implemented in the *robumeta* package in R. This method accounts for the clustering of effect sizes within studies.

We ran analyses separately for official measures and victim reported measures of recidivism. These were run separately and combined for random assignment studies and quasi‐experimental studies. A moderator analyses compared the mean effect size between the random assignment and quasi‐experimental studies within each outcome type.

#### 4.4.3 Treatment of qualitative research

This review did not synthesize any existing qualitative research in the area of intimate partner violence.

## 5 RESULTS

### 5.1 Description of studies

#### 5.1.1 Results of the search

The above process identified 21,329 titles and abstracts (excluding duplicates). A total of 124 studies were retrieved in their entirety for further scrutiny, which resulted in the identification of two new studies. Of these, a total of four experimental studies and seven quasi‐experimental studies were identified as meeting the eligibility criteria. The study descriptors including year of publication, treatment type, number of treatment sessions and weeks, nature of the comparison group, and sample description are reported in Table 1. All 11 studies were conducted in North America with the exception of two in Canada and one in Australia. All studies except one were published in peer reviewed journals, although technical reports were also available for four studies (see reference list). When there was conflicting information between the two sources, data from the nonpublished technical report was used because these reports typically provided more detailed information. No studies were excluded due to missing data necessary to compute an effect size.

**Table 1:** Study descriptors

Author/year	Program type	Program weeks/sessions/hours	% Completing program	Control type	Sample
*Random assignment studies*					
Davis et al. (2000) (26 week program)	Duluth Model (26 weeks)	26/26/39	27	Community service	Convicted batterers; judge, prosecutor and defense agree to treatment mandate
Davis et al. (2000) (8 week program)	Duluth Model (8 weeks)	8/16/39	67	Community service	Convicted batterers; judge, prosecutor and defense agree to treatment mandate
Dunford (2000) (conjoint)	Cognitive‐behavioral conjoint group	52/32/48	71	No treatment	US Navy couples; substantiated physical abuse
Dunford (2000) (men's group)	Cognitive‐behavioral men's group	52/32/48	71	No treatment	US Navy husbands; substantiated physical abuse
Dunford (2000) (rigorous monitoring)	Rigorous monitoring (monthly individual counseling)	52/12/12	71	No treatment	US Navy husbands; substantiated physical abuse
Feder and Forde (2000)	Duluth Model	26/26/39	66	No treatment	Convicted batterers
Labriola et al. (2002)	Batterer program plus judicial monitoring	26/26/32.5	Missing	Judicial monitoring	Misdemeanor domestic violence; prosecutor and defense agree to referral to batterer and judicial monitoring
*Quasi‐experimental studies*					
Blatch et al. (2016)	Duluth and cognitive‐behavioral	10/20/45	Missing	No treatment	History of domestic abuse; high/medium risk (LSI‐R)
Chen et al. (1989)	Time Out program	Missing/missing/missing	63	No treatment	Convicted batterers
Dutton (1986)	Cognitive‐behavioral program	16/16/48	100	No treatment	Convicted batterers
Gordon and Moriarty (2003) (mandated vs. not)	Duluth Model	22/22/missing	61	No treatment	Convicted batterers
Harrell (1991)	Cognitive‐behavioral	10/10/15	76	No treatment	Misdemeanor charge of offense against a female partner; suspended prosecution
Palmer et al. (1992)	Psychoeducational	10/10/15	70	No treatment	Convicted batterers
Syers and Edleson (1992)	Court‐ordered treatment	Missing/missing/missing	Missing	No treatment (not mandated to treatment)	All batterers having police contact who could be followed for 12‐months

#### 5.1.2 Description of included studies

All eleven studies evaluated a psychoeducational or cognitive behavioral approach, or some mix of the two approaches targeted at the batterer and delivered in all‐male group settings (see Tables 1 and 2). One study, Dunford (2000), tested three intervention types relative to a no‐treatment control: a cognitive behavioral group for male batterers, a cognitive behavioral group targeted at the male batterer, but conducted in conjoint groups with the batterer's partner, as well as a no‐program, but rigorously monitored intervention involving monthly individual counseling sessions for the male batterer. Additionally, Davis et al. (2000) examined two delivery variants of the Duluth model, an 8‐week version with 16 sessions and a 26‐week version with 26 sessions. The total group time was the same for both at 39 hours and the content was the same. The treatment variations within Dunford (2000) and Davis et al. (2000) were treated as separate evaluations in the analysis (see Table 1). In all but three of the studies (i.e., Chen et al., 1989; Dunford, 2000; Harrell, 1991) the program intervention was accompanied by probation, although in one of these studies Chen et al. (1989), it seemed likely that probation was a factor as well and is also likely true for many of the participants in the other studies. The participants in the Labriola et al. (2002) study received a conditional discharge.

**Table 2:** Method features and risk‐of‐bias

Author/year	Research design	Treatment/control sample sizes	How analyzed	Risk of selection bias	Attrition (official measures) (%)	Selective reporting of outcomes
*Random assignment studies*						
Davis et al. (2000) (26 week program)	Random assignment	129/186	Intent‐to‐treat	Low‐risk	100	Probably No
Davis et al. (2000) (8 week program)	Random assignment	61/186	Intent‐to‐treat	Low‐risk	100	Probably No
Dunford (2000) (conjoint)	Random assignment	146/144	Intent‐to‐treat	Low‐risk	100	No
Dunford (2000) (men's group)	Random assignment	160/144	Intent‐to‐treat	Low‐risk	100	No
Dunford (2000) (rigorous monitoring)	Random assignment	168/144	Intent‐to‐treat	Low‐risk	100	No
Feder and Forde (2000)	Random assignment	230/174	Intent‐to‐treat	Low‐risk	99	No
Labriola et al. (2002)	Random assignment	202/218	Intent‐to‐treat	Low‐risk	100	No
*Quasi‐experimental studies*						
Blatch et al. (2016)	Quasi‐experimental (propensity score matching)	953/953	Intent‐to‐treat	High‐risk: direction not predictable	100	No
Chen et al. (1989)	Quasi‐experimental (instrumental variable analysis)	120/101	Treated	Low‐risk	92	No
Dutton (1986)	Quasi‐experimental (untreated due mostly to no space in treatment program)	50/50	Treated	High‐risk: bias favors treatment	100	No
Gordon and Moriarty (2003) (mandated vs. not)	Quasi‐experimental (statistical controls for baseline differences)	132/116	Treated	High‐risk: bias favors treatment	100	No
Harrell (1991)	Quasi‐experimental (statistical controls for baseline differences)	171/177	Treated	High‐risk: bias favors treatment	52	No
Palmer et al. (1992)	Quasi‐random (assigned to treatment if new group was to start within 3 weeks)	30/29	Treated	Unclear risk	95	No
Syers and Edleson (1992)	Quasi‐experimental	37/87	Treated	High‐risk: bias favors treatment	61	No

The treatment length ranged from a minimum of eight 2‐h sessions (Chen et al., 1989) to a maximum of 32 sessions over the course of a year (Dunford, 2000). Treatment length information was not provided by Syers and Edleson (1992). The contact time (programming hours) ranged from a low of 15 to a high of 48. However, actual contact hours are often substantially less given that attendance is a major challenge for these programs. For 11 of the 14 treatment‐comparison contrasts, the percentage of participants completing the program, often defined as a percentage of sessions attended (e.g., 80%), was both reported and often problematically low. These percentages ranged from a low of 27% (Davis et al., 2000; 26‐week program) to 71% (Dunford, 2000). The study by Dutton (1986) had 100% meeting completion as that sample was restricted to those participants who actually completed the program, creating potentially biased results favoring the program condition.

The primary distinction between the experimental or program group and the control or comparison group was that those in the former participated in the BIP program and those in the latter did not. Other sanctions, such as probation or judicial monitoring, were generally the same for both groups. The only exception was Davis et al. (2000) where the program group received the Duluth model and the control group received an equal number of community service hours as the alternative. Most studies provided very limited information on the nature of the comparison condition other than that they were not mandated to the BIP.

All but one of the eleven studies used a general civilian population of batterers who were facing or had faced court prosecution for intimate partner violence. The one exception, Dunford (2000), used men living on a Navy base for whom an incident of intimate partner violence had been established and a referral made to the program.

In five studies the generalizability of the sample to the general intimate partner violence offender population was questionable due to restricted conditions used for inclusion into the sample. For example, Davis et al. (2000) only included individuals for whom the courtroom workgroup (judge, prosecutor, and defense attorney) as well as the batterer agreed to the court mandate to the intervention versus another nonjail alternative like community service. This, as the researchers noted, led to a pool of more highly motivated offenders than is typically found in the generalized batterer population. We suspect that the sample in the Palmer et al. (1992) study was also highly restrictive given that the resulting sample size was small despite the large jurisdiction from which it was pulled and the long‐time frame for data collection. However, limited information is provided by Palmer et al. (1992) on this detail. In the Dunford study (2000), the men were all living on a naval base with their families and therefore may represent a group with a higher stake in conformity than is true of other batterer samples. In Syers and Edleson (1992) quasi‐experimental study, only those men who could be followed 6‐ and 12‐months postinitial police visit were included in the study. This restriction makes it less likely that more marginal batterers would be included in their study, thereby biasing results in favor of finding beneficial treatment effects. Finally, in Labriola et al. (2002), the judge, prosecutor, and defense attorney all had to be in agreement regarding the conditional release of the offender. The implication of these restrictions is that the findings from this systematic reviewer are likely applicable to a subset of batterers who are more motivated and have a higher stake in conformity (as indicated by having more steady employment, relationships, and living arrangements) than the broader population of men who engage in intimate partner violence.

### 5.2 Risk of bias in included studies

Several methodological characteristics related to the credibility of the findings in terms of the effectiveness of the batterer program(s) were coded (see Table 2). At the design‐level, four of the studies representing seven treatment‐comparison contrasts used a random‐assignment design. One of these, Palmer et al. (1992), was coded as a random‐assignment design in the prior version of this review. However, the design is more accurately described as quasi‐random because selection into the program or control was determined by the timing on when the next program would start. All four of the random assignment studies were judged as low‐risk for selection bias, as there was no evidence of problems with randomization or obvious baseline differences. One of the quasi‐experimental designs, Chen et al. (1989), was also judged as low‐risk for selection bias. This study used an instrumental variable analysis method where the instrument for estimating the treatment effect was based on a model that predicted a judge's probability of mandating someone to a BIP. This is a high quality quasi‐experimental method that should produce an unbiased estimate of the treatment effect.

Two studies, Palmer et al. (1992) and Dutton (1986), used a quasi‐random assignment procedure (a form of a wait‐list). While these designs can be low risk of bias, Palmer et al. provided no information on the equivalence of the groups at baseline. As such, we judged this design to be of unclear risk of selection bias. Dutton (1986) provided baseline equivalence data and while the groups were highly similar on most variables, the control group had a higher number of prior assaults and a slightly higher rate of unemployment, suggesting a possible selection bias in favor of the treatment group performing better when compared to the control group. Furthermore, this study only included men who completed treatment in the treatment group, creating another potential source of selection bias in favor of finding the treatment effective. As such, we judged this study to be at high‐risk for selection bias in favor of the treatment group. The remaining quasi‐experiments used some form of statistical controls for baseline, either in the form of propensity score matching (Blatch et al., 2016) or multiple regression analysis (Gordon & Moriarty, 2003; Harrell, 1991; Syers & Edleson, 1992). These were all judged as being at high‐risk for selection bias given the possibility of omitted‐variable bias. With the exception of the propensity‐score matching study by Blatch et al. (2016), our assessment was that the likely direction of the bias favored the treatment condition. For Blatch et al. (2016), we were not able to make any assessment of the likely direction of any selection bias.

Missing outcome data is generally not an issue for these studies given the reliance on official measures of repeat offending as the primary outcome. However, missing outcome data was a significant issue for two studies: Harrell (1991) and Syers and Edleson (1992). Most of the random assignment studies performed an intent‐to‐treat analysis whereas most of the quasi‐experimental designs estimated the effect of treatment on the treated. Selective reporting of outcomes was not judged to be an issue in these studies.

Unfortunately, the use of official measures of recidivism creates a potential risk‐of‐bias related to outcome measurement for all of these studies. This was not something we coded at the study‐level as it affects all outcomes based on official measures. The potential bias is the possibility that assignment to the batterer program may affect the probability of observing repeat offending if it does occur. The basis for this concern is that the primary way for the police, court, or probation officers to learn of repeated abuse is from a phone call by the victim or sometimes a neighbor. If a batterer is angry at being required to participate in the program, then their partner may be less likely to report a new incident of abuse as she may be fearful of her partner's wrath. The finding of smaller and generally null effects for victim self‐reports of abuse made directly to researchers under a condition of confidentiality reinforce this possibility. However, the victim reported measures suffer from high attrition with most studies only able to get roughly 50% of the victims to participate in a follow‐up interview. Thus, we assess all of these studies as being at high‐risk of bias related to outcome measurement.

### 5.3 Synthesis of results

The effect sizes were analyzed separately by outcome type (official reports and victim reports) and by design type (random assignment designs and quasi‐experimental designs with a no‐treatment comparison group). The separate analysis of effects by design type was the sole statistical method used to incorporate risk‐of‐bias in the synthesis of effects across studies. Table 3 presents the random‐effects mean effect size, 95% confidence interval (CI), and homogeneity statistic (*Q, I* ^2^, and *τ* ^2^) for both outcome types and each design type. The combined sample size across these studies was 4824.

**Table 3:** Results of meta‐analyses of official measures and victim measures of repeated intimate partner violence for randomized and quasi‐experimental studies

		95% CI						
Analysis	Odds ratio	Lower	Upper	*Q*	*p* of *Q*	*I* ^2^	τ^2^	No. of effect sizes
Official measures^a^								
Randomized studies	0.79	0.49	1.28	3.832	0.699	0%	0.00	7
Quasi‐experimental studies	0.54	0.24	1.22	18.929	0.004	68%	0.792	7
Randomized and quasi‐exp.	0.67	0.43	1.05	23.165	0.040	44%	0.305	14
Victim reported measures^b^								
Randomized studies	0.99	0.74	1.32	1.031	0.984	0%	0.00	7
Quasi‐experimental studies	1.76	0.50	6.14					1
Randomized and quasi‐exp.	1.02	0.77	1.35	1.808	0.970	0%	0.00	8
All effect sizes^c^	0.74	0.47	1.16			48%	0.26	140

*Note*: An odds ratio <1 favors the treatment group. Random‐effects models estimated a priori using Dersimonian‐Laird estimator for the random effects variance component, *τ* ^2^. For three analyses, τ^2^ equaled zero. Thus, those models converged to fixed‐effect models. Only one effect estimate was available for victim reported measures from a quasi‐experimental study.

^a^ Moderator analysis test of the difference between the mean for random and quasi‐experimental studies for official measures was not statistically significant (*Q*_between_ = 0.427, df = 1, *p* = .5134).

^b^ Moderator analysis test of the difference between the mean for random and quasi‐experimental studies for victim reported measures was not statistically significant (*Q*_between_ = 0.778, df = 1, *p* = .3778).

^c^ All 140 effect sizes across the 11 studies, 14 treatment‐comparison contrasts were used in this analysis. The analysis clustered the effect size by the study identifier (*n* = 11) and estimated robust *SE*s using the method developed by Hedges et al. (2010).

#### 5.3.1 Official reports

Official reports were either official complaints made to the police that may or may not have resulted in an arrest, or actual arrests for intimate partner violence. Five studies reported multiple official measures of repeated intimate‐partner violence. Preferences was given to earlier measures that reflected less progression through the criminal justice system, such as calls to the police over arrests, arrests over convictions, and so forth. We also gave preference to longer follow‐up periods over shorter periods and, for quasi‐experimental studies, effect sizes were adjusted for observed baseline characteristics. Davis et al. (2000), representing two treatment‐comparison contrasts, reported results for a 6‐ and 12‐month follow‐up, posttreatment. The 12‐months follow‐up for each treatment‐comparison contrast was used. Syers and Edleson (1992) also reported recidivism rates for 6‐ and 12‐month follow‐up, posttreatment. Furthermore, they reported an estimate of the treatment effect at 12‐months adjusted for observed baseline differences. The latter was used. Two effect sizes Harrell (1991) reported used two official measures, one based on domestic violence calls to the police and the other on domestic violence charges. The former were used. Gordon and Moriarty (2003) reported data on domestic violence arrests and convictions. The former was used. Labriola et al. (2002) reported data at 12‐ and 18‐months follow‐up. We used the latter.

**Figure 1:**
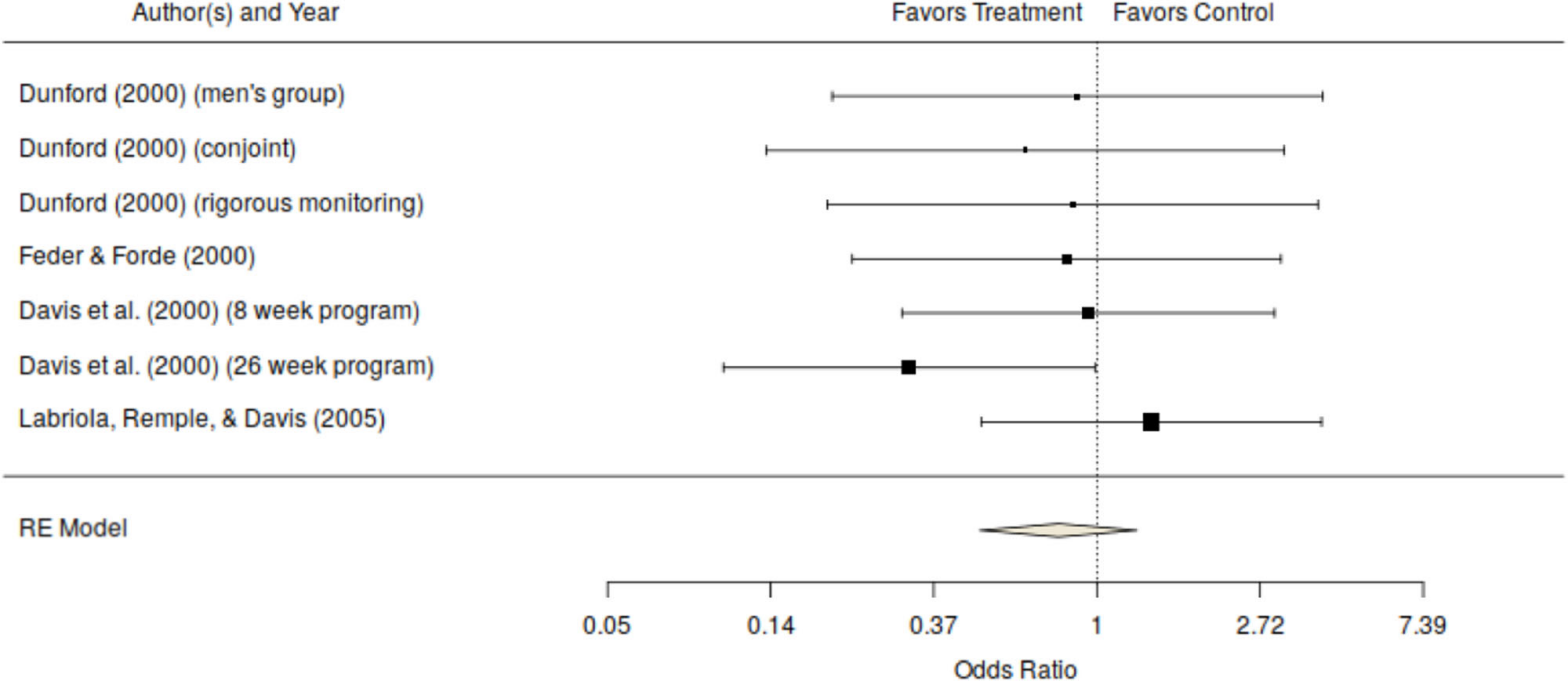
Official measures of repeat offending odds ratio and 95% confidence interval for random assignment studies

As can be seen in Table 3, the mean effect size for the randomized studies across these seven comparisons from four independent studies was moderate in size and favored the treatment group. However, it was not statistically significant (odds ratio, 0.79; 95% CI [0.49–1.28], *k* = 7). There is a lack of evidence of heterogeneity (*Q* = 3.832, *df* = 6, *p* = .699, *I* ^2^ = 0%) although the *Q* statistic is known to be statistically under‐powered when the number of studies is small. Figure 1 shows the forest plot for these effects. This figure shows that most of the effects are near the null value with only one effect that is of a substantively meaningful size in the desired direction (the Davis et al., 2000, 26 week program). Even with this outlier, the pattern of evidence in this figure suggests that these programs are unlikely to produce substantively meaningful effects.

The one large beneficial effect is also somewhat puzzling. The effect was for the 26‐week version of the program in the Davis et al. (2000) study. The effect for the same program delivered over 8 weeks showed essentially null results. However, treatment completion was higher for the 8‐week program than the 26‐week program. It is entirely possible that the difference between these two conditions is simply chance variation, as the difference itself is not statistically significant. However, we would expect the group with a higher percentage of men completing the program to be associated with the large effect, yet treatment effects were higher for the men assigned to the 26‐week program. This raises the possibility that this sole significant and meaningfully beneficial effect is simply a spurious result. Alternatively, Feder and her colleagues (Feder & Dugan, 2002; Feder & Forde, 2000) speculated that these results were more consistent with a conclusion that supervision, and not treatment, resulted in the groups' differences in rates of re‐assault.

In fact, this odd finding led Davis and his collaborators to re‐analyze the data from their study (Maxwell et al., 2010). Again, men in the 8 week group and those in the 26 week group received the same number of hours of treatment. However, men in the 8 week group were more likely to complete the program yet less likely to desist in their use of intimate partner violence than the men in the 26 week program. Additionally, they found no evidence in cognitive changes in men in either treatment group contrary to what one would expect if treatment had been effective. This led to their new conclusion that it was court supervision and not treatment that led to the differences in recidivism. That is, men under court control for longer periods of time (the 26 week treatment group) did better than those under a shorter period of supervision (the 8 week treatment group) despite the fact that the latter group was more likely to have completed the treatment sessions. While this is important background information on this study, this re‐analysis was not eligible as part of our synthesis as it included the treatment refusers in the control group.

**Figure 2:**
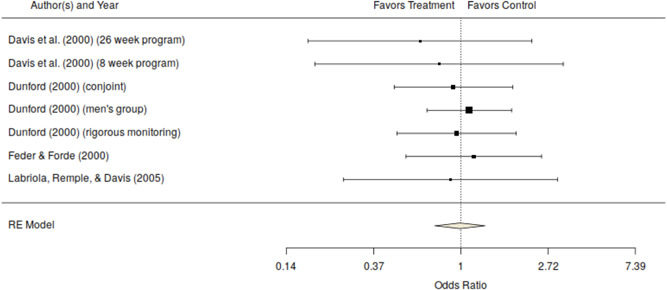
Official measures of repeat offending odds ratio and 95% confidence interval for quais‐experimental studies

For the quasi‐experimental studies, the mean odds ratio for official measures of repeat offending (odds ratio, 0.79; 95% CI [0.49–1.28], *k* = 7). This effect is in the desired direction and of a substantively meaningful size (i.e., it would represent a recidivism reduction from 50% to 35%), but it is not statistically significant and has a confidence interval that extends well past the null value (see Table 3). As can be seen in Figure 2, the individual effects are highly variable (heterogeneous) with a mix of effects favoring the treatment (5) and favoring the no treatment control group (2). The effect size from Dutton (1986) is the largest but is based on a modest sample size (50 per group) and the treatment group was restricted to completers. The confidence interval for this study is wide, reflecting uncertainty in the effect. This is also the only effect that is statistically significant. While the pattern of evidence favors the treatment group, the mixed nature of the evidence and nonsignificance of the mean odds ratio preclude any strong conclusions about effectiveness. Furthermore, most of these studies were judged as at high‐risk of selection bias typically favoring the treatment group.

The overall effect for official measures of repeat offending across both randomized and quasi‐experimental studies was also not statistically significant (odds ratio, 0.67; CI [0.43–1.28], *k* = 14). The test of the difference between the mean effect size for these two types of studies was also not statistically significant (*Q*_between_ = 0.427, df = 1, *p* = .5134).

#### 5.3.2 Victim reported outcomes

**Figure 3:**
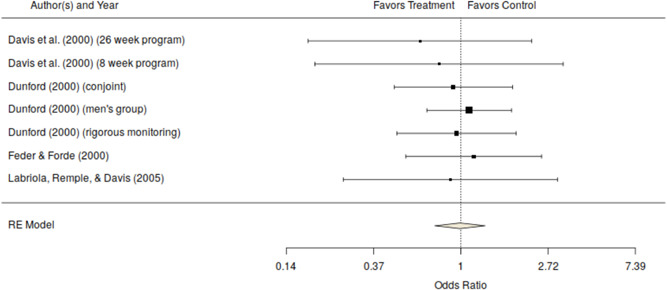
Victim measures of repeat offending odds ratio and 95% confidence interval for randomized studies

**Figure 4:**
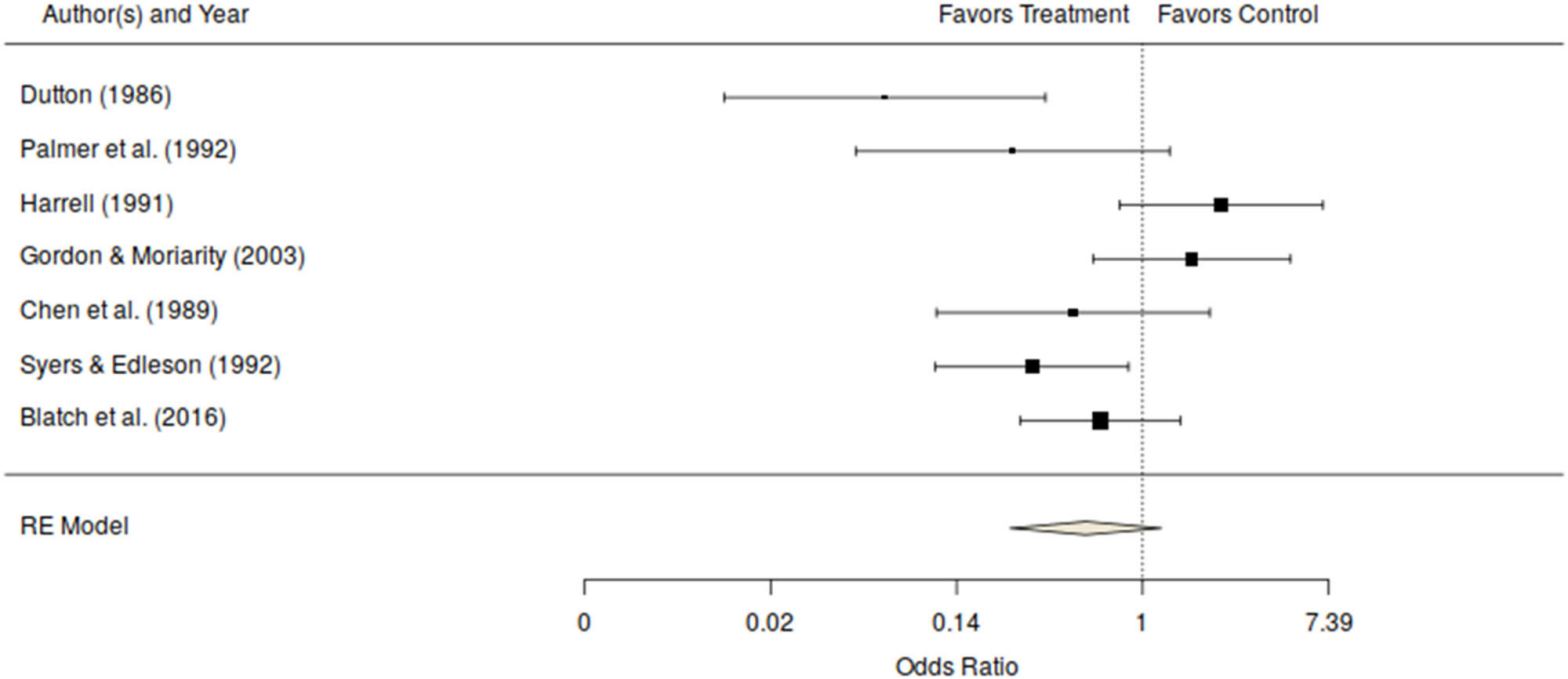
Victim measures of repeat offending odds ratio and 95% confidence interval for quasi‐experimental studies

A concern with official measures is that they may not accurately reflect the amount and severity of on‐going violence. Research consistently indicates that official reports capture only a small fraction of intimate partner violence (Buzawa & Buzawa, 2017; Dutton, 1988; Myhill & Johnson, 2016; Policastro & Payne, 2013; Straus, 1991; Tjaden & Thoennes, 2000). As discussed earlier, there is also concern of potential bias in these measures if the intervention affects the likelihood of reporting repeat offending to the police or other justice officials. As such, the victim is viewed as the best source for information on the offender's continued abuse. Given that, we turn our attention to the seven estimates based on victim reports of abuse or repeat offending from random assignment studies. All of the random assignment studies measured the victim's reports of their partner's abusive behavior using either the standardized CTS or the modified CTS (CTS2) (Straus et al., 1996). One of the quasi‐experimental studies also measured the victim's report of their partner's abusive behavior using a measure similar to the CTS. For purposes of analysis, we coded all reported subscales and averaged the multiple effect sizes within each treatment‐comparison contrast. Thus, the effect size used in Table 3 and Figures 3 and 4 represent the mean effect across subscales of the CTS/CTS2 for the comparison of interest. As shown in Table 3, the mean odds ratio for victim reports in studies using a random assignment design equaled the no effect value of roughly 1 (odds ratio, 0.99; 95% CI [0.74–1.32], *k* = 7) and was homogeneous across studies (see also Figure 3). The effect for the single quasi‐experimental study with a victim reported outcome favored the no treatment comparison group, although this effect was not statistically significant (see Figure 4) (odds ratio, 1.76; 95% CI [0.50–6.14], *k* = 1). The overall effect for victim reported measures of repeat offending across both randomized and quasi‐experimental studies was also not statistically significant (odds ratio, 1.02; CI [0.77–1.35], *k* = 8). The test of the difference between the mean effect size for these two types of studies was also not statistically significant (*Q*_between_ = 0.778, df = 1, *p* = 0.3778). As can be seen in Figure 3, the effects of court mandated programs on victim reported outcomes are mostly near the null value. This suggests that these programs are not having the desired effect, at least based on victim's reports of on‐going violence. Unfortunately, this measure suffers from high levels of missing data. Not all victims are willing to be interviewed at follow‐up and some are no longer living with the perpetrator, making it impossible for them to assess change in his behavior.

#### 5.3.3 Sensitivity analysis

The above analyses of official measures of repeat offending are based on selected effect sizes. However, a total of 140 effect sizes were calculated across all studies, including victim reported measures. Most of these (87) came from a single study (Dunford, 2000), with all other studies having between 1 and 19 calculable effect sizes. The median number of coded effects per study was 4. To ensure that the above analyses are not biased as a result of the particular effect size selected for analysis for each study, we ran an alternative model using all effect sizes. To deal with the dependencies created by having multiple effect sizes based on the same individuals, we used the robust *SE*s method (Hedges et al., 2010), clustering on the 14 treatment‐comparison contrast. We also ran the analyses clustering at the study level (11 clusters). The latter is shown in Table 3, but the two models were highly similar. The mean odds ratio for this model favors the treatment group and is roughly the same as the model of official measures of repeat offending for random assignment studies (0.74 compared to 0.79). This mean odds ratio is not significant (95% CI [0.47–1.16]). Using all odds ratios leads to the same general conclusion as the individual analyses reported above, which is that these BIPs do not seem to lessen the likelihood of repeat violence.

Another potential issue with the above analyses was that two of the treatment conditions from the Dunford (2000) study are unlike the programs examined in all other studies, the conjoint condition and the intensive monitoring condition. This was the only study to examine a conjoint program model and we are not aware of any wide‐spread use of such programs. The intensive monitoring program involved monthly counseling and did not involve any manualized or structured programming component. This study did include a more typical men's only cognitive‐behavioral program that is similar to the programs assessed in the other studies. Thus, we re‐ran the analysis for official and victim reported measures for the random assignment studies exclusively including the men's only group from Dunford. We also combined the data for the 8‐ and 26‐week conditions into a single condition and recomputed the effect size. These analyses reinforce the general conclusion that the evidence does not support the conclusion that these programs are effective. Under this re‐analysis, the mean odds ratio for official measures of repeat offending was 0.83 compared to 0.79 in the first analysis, with a 95% CI of 0.46–1.49. For victim reported measures, the mean odds ratio was 1.04 (essentially null), with a 95% CI of 0.41–1.51.

These sensitivity analyses were posthoc and not planned as part of the protocol for this review.

#### 5.3.4 Publication selection bias

Seven of the eleven studies were published. We explored publication selection bias through several analyses. First, we ran a moderator analysis that compared the odds ratios from published versus unpublished studies. This difference was not statistically significant (see Supporting Information Appendix B). The mean odds ratio for published studies represented a larger beneficial effect (0.52) than the mean odds ratio for unpublished studies that had a mean odds ratio near the null value of 1 (0.98). This is suggestive of publication selection bias, which suggest those studies finding treatment effectiveness may be more likely to be published and easily found by our search strategy. Second, we performed a trim‐and‐fill analysis on the 14 odds ratios used in the analyses of official measures of repeat offending (random assignment and quasi‐experimental studies). This did not detect any evidence of publication selection bias. Third, we generated two funnel plots. The first was on the 14 odds ratios used in the prior trim‐and‐fill analysis (see Supporting Information Appendix B) and the second was on all 140 odds ratios (see Supporting Information Appendix B). Both of these in our assessment show some evidence of missing effects in the lower right region (e.g., null or harmful effects from small studies). Finally, an Egger's test for asymmetry on the funnel plot was suggestive of some asymmetry with a *p* = 0.10. Thus, we would judge this body of evidence to be at slight risk of publication selection bias. That is, the mean odds ratios presented in Table 3 may be somewhat biased in favor of the BIPs. This adds additional concern that these programs may not be effective at reducing repeat physical violence against an intimate partner.

## 6 DISCUSSION

### 6.1 Summary of main results

This systematic review was based on 11 experimental and quasi‐experimental studies representing 14 treatment‐comparison contrasts. All of the studies assessed the effect of mandated BIPs relative to a no‐treatment or routine‐treatment approach for men facing or convicted of misdemeanor intimate partner violence charges. The BIP in these studies was either based on the Duluth model or a similar psychoeducational program with a feminist orientation, a cognitive‐behavioral orientation, or a mix of the two.

The evidence from our meta‐analysis raises doubts regarding the effectiveness of these programs. On official measures, those mandated to the treatment group exhibited lower rates of repeat intimate partner violence than those not mandated, but the overall mean effects were not statistically significant for both randomized experiments and quasi‐experiments. In contrast, however, the overall mean effects were either no different or favored the comparison (no treatment) condition for victim reported measures of repeated abuse for randomized experiments and one quasi‐experiment, respectively. The victim reported outcomes are surprisingly homogeneous and centered around the null value of no effect.

This leads to an equivocal conclusion. While the evidence does not support a conclusion favorable to the effectiveness of these programs it is also insufficient to establish that the programs are ineffective or harmful. The confidence intervals around the mean effects are large, suggesting meaningful uncertainty regarding the true effectiveness of these programs. Furthermore, the methodological weakness, discussed more fully below, preclude drawing strong conclusions. However, the pattern of evidence across these studies is inconsistent with what we would have expected from a program that produces consistent meaningful reductions in repeated abuse across the variations of implementation and context present in these studies.

### 6.2 Quality and completeness of the evidence

A major quality concern with effectiveness trials is selection bias or differences in the outcome that simply reflect differences in the characteristics of the groups. Roughly half of these studies were judged to be at low‐risk of selection bias either given the use of a true experimental design with random assignment to conditions or the use of a high quality quasi‐experimental design with good control for the selection mechanism (i.e., instrumental variable analysis). Counter‐intuitively, the effects favored the comparison for two of the three quasi‐experimental designs judged to be biased in favor of the treatment group.

A risk‐of‐bias concern of our is that the official measures of repeat offending may be biased in favor of the treatment. Official measures are dependent on a victim's willingness to file a complaint or call the police. This raises the possibility that assignment to court‐mandated treatment versus a no‐treatment control group may deferentially affect the victim's willingness to contact criminal justice officials when future abuse occurs (Cook and Campbell, 1979, refer to this as an instrumentation by selection effect). A victim may not report her partner's abuse for a number of reasons. This includes the possibility that she might prefer to see her partner continue in treatment where she believes it will eventually lead to changes in his abusive behavior rather than take the risk of reporting his continued abuse and see him go to jail. Alternately, a victim may resent the criminal justice system's intrusion into her life in the form of mandating a treatment that she must pay for. Most programs require the abuser to pay for the treatment and by extension that means the family pays for the treatment (Zorza, 2003). If the treatment is viewed by a victim as ineffective, it may make her critical and suspicious of the system and less likely to cooperate in the case of reporting future incidences of abuse. We have no empirical evidence that this bias in official measures exists. However, the dependence on official reports about the behavior of the victim allows for the plausibility that the different rates noted between batterers in the treatment and comparison conditions may reflect a measurement artifact and not a genuine, albeit nonsignificant, treatment effect. Our concern regarding the bias in official measures is strengthened by the differentiated effects between official reports and victim reports. Victim reported measures showed either a null effect of these programs (random assignment studies) or a harmful effect of these programs (quasi‐experiments), although again these were not statistically significant and had large confidence intervals.

The victim is usually viewed as the best source for information on the offender's continued abuse. In all of these studies, victim reports of abuse were assessed using either a version of the CTS or a measure highly similar to this scale. This scale is less likely to be affected by the issues raised regarding official reports of continued abuse, provided that the victim is assured of the confidential nature of her responses. In all of these studies, the victim‐reported information was collected by the researchers and not by anyone directly affiliated with the criminal justice system. Unfortunately, the percentage of victims responding to follow‐up surveys in these studies is low, raising the possibility of nonresponse bias. The main concern is differential nonresponse bias: the possibility that the victims that did not provide outcome data may differ in meaningful ways from those in the control group who did not provide outcome data. Thus, the absence of an effect for the victim report measures may reflect that the programs are truly ineffective or, alternately, that there is a positive or negative effect that is masked by differential nonresponse.

The problem of high rates of victim attrition becomes critical in light of research indicating that certain victims of intimate partner violence are more likely to be lost in the research follow‐up than others. This research strongly suggests that female victims of intimate partner violence who are more difficult to retain in follow‐up research are both more marginal and more likely to be more frequently and severely abused (Sullivan et al., 1996). There is also research that indicates that men who are more marginal are both less likely to obey a court‐mandate to treatment and more likely to continue to abuse their partners (Feder & Dugan, 2002). If we can assume that more marginal women are more likely to be partnered with more marginal men, than the need for maintaining contact with a high percentage of victims when assessing the effectiveness of these intimate partner violence abatement programs becomes even more apparent. This may be important to the extent that some research has indicated that factors associated with the abuser's stake in conformity is associated with the likelihood that an intervention will be successful in reducing subsequent violence (Berk et al., 1992; Sherman, 1992). At best, this nonresponse problem reduces the generalizability of the findings from victim reported outcomes to a subset of the intimate partner violence offender population. At worst, there may be differential loss of these marginal women from the treatment and control groups, producing bias in the findings.

Another research quality concern relates to generalizability. We judged two studies, Davis et al. (2000) and Palmer et al. (1992), as having samples that were restricted in a manner that reduced the generalizability of their findings to a general batterer population. One of these was also the only random assignment study reporting meaningfully beneficial results of BIPs. The study was also one of the larger effects among the quasi‐experiments. This may suggest that BIPs work for a selected (presumably more motivated) subset of offenders. Unfortunately, there was an insufficient number of effect sizes to explore this through a formal moderator analysis. Furthermore, the evidence on this issue is weak for two reasons: (1) we do not actually know the motivation levels of the men in the different studies, and (2) the Davis et al. study had inconsistent results across two similarly motivated groups receiving the same intervention, differing only in the number of weeks over which the program was spread. The generally smaller effects for the representative samples only reinforces the general conclusion that these programs may not be effective at reducing future intimate partner violence.

The systematic search for this review was last updated in February of 2018. We are not aware of any newer studies that would meet the eligibility criteria for this review. Furthermore, our search failed to identify any studies conducted within the past decade. New research in this area has focused on comparing two different types of BIPs, such as cognitive‐behavioral programs with and without a motivational interviewing omponent. These head‐to‐head comparisons have recently been meta‐analyzed (Santirso et al., 2020) but are not eligible for this review. Thus, while newer eligible studies may have been conducted, it is unlikely that there are enough of such studies to change this review's results fundamentally.

### 6.3 Agreements and disagreements with other studies or reviews

Our findings are somewhat different from those of Babcock et al. (2004). They concluded, based on their meta‐analysis, that these programs have a small but positive effect on abusive behavior. There are several differences between the methods employed in our respective meta‐analyses that may account for the differing conclusions. Primarily, Babcock et al. (2004) included treatment drop‐out designs along with other quasi‐experimental and experimental designs. The prior version of this review also included drop‐out designs, but analyzed them separately and showed that they provide very large and positive estimates of treatment effects. As we argued in our prior version of this review, there is strong reason to doubt the outcomes from those studies. Offenders mandated to a BIP who do not complete the program are likely to be different in important ways from offenders who complete the program. For instance, those who fail to complete these programs might be less motivated to change than those offenders who are successful in finishing treatment. If one looks only at experimental studies, results from both meta‐analyses are fairly consistent. Babcock et al. reported a Cohen's *d* effect size of 0.12 when using official reports (fixed effects 95% CI of 0.02–0.22). This is somewhat smaller than our overall mean effect which was equivalent to a Cohen's *d* for official reports based on experimental studies (our odds ratio of 0.72 converts to a Cohen's *d* of 0.18).

A systematic review that did not include a meta‐analysis of the findings by Cluss and Bodea (2011) concluded that “there is no solid evidence for either the effectiveness or relative superiority of any current group interventions” for batterers (p. 10). This is largely consistent with our findings, but stated more forcefully. This review also agrees with the prior version of this systematic review, although the addition of two studies, including one random assignment study, only reinforces the conclusion that there is insufficient evidence to support the effectiveness of these programs.

A recent meta‐analysis by Cheng et al. (2019), agreeing with this review, failed to find evidence of effectiveness across randomized controlled trials but did, in contrast to this review, find evidence of possible effectiveness across the collection of quasi‐experimental studies. Similarly, a review by Smedslund et al. (2011) of both voluntary and court‐ordered programs found no evidence of an effect for cognitive‐behavioral programs for men who physically abuse their partners.

The most optimistic recent review is by Arce et al. (2020). This review concluded that cognitive‐behavioral therapy programs were effective but that those based on the Duluth model were not, and that the latter potentially have negative effects. However, as discussed in the background section, this review used methods that raises concerns regarding the credibility of these findings. These concerns includes failing to maintain statistical independence across effect sizes in the analysis, thus overstating statistical significance, and calculating effect size estimates that did not include the findings from the control group. A narrative review of 39 studies of BIPs by Eckhardt et al. (2013) also arrived at a more optimistic assessment of the effectiveness of these programs. This study included a broad mix of design types and programs for victim‐survivors as well as batterer‐specific programs. The conclusions specific to the latter were equivocal with the evidence base being described as providing “very mixed conclusions regarding BIP effectiveness” (p. 220). However, they expressed optimism for newer programs that focus on motivation and readiness for change. Murphy and Ting (2010) drew a similar conclusion arguing that newer approaches that focus on enhancing program attendance and motivation for change “have yielded consistently encouraging results” (p. 26) on therapeutic variables such as attendance, compliance with program tasks, etc.

Two meta‐analyses support the inference that motivational interviewing enhances the value of these programs. For example, Santirso et al. (2020) meta‐analyzed 12 randomized controlled trials that compared a standard batterer intervention to the same program with a motivational component. They concluded that the, “Results indicated that IPV interventions that incorporated motivational strategies were significantly more effective in increasing the intervention dose and reducing dropout than interventions without motivational strategies” (p. 175). Soleymani et al. (2018) arrived at a similar conclusion based on a meta‐analysis of five studies. This meta‐analysis showed that motivational interviewing as a pretreatment adjunct improved level of engagement, sessions attendance, and homework compliance. In terms of physical and psychological intimate partner violence and official recidivism, Santirso et al. (2020) failed to find a statistically significant benefit.

## 7 AUTHORS' CONCLUSIONS

### 7.1 Implications for practice and policy

The findings from this meta‐analysis combined with the caveats above raise questions as to the value of these programs. While additional research is needed, the meta‐analysis suggests that court‐mandated treatment to group‐based programs for misdemeanor intimate partner violence offenders is unlikely to reduce assaultive behaviors. While we cannot definitively conclude that these programs don't work, the pattern of evidence is inconsistent with what we would expect if they produced meaningful reductions in repeat offending across natural program variations and settings.

Intervening in the lives of others is a risky business, particularly when the individuals participating in the social intervention are mandated by a court of law to do so. As such, it is incumbent upon us to ensure that we are not inadvertently making things worse for those we are seeking to help. At this point the existing evidence cannot ensure that these programs are, in fact, helpful and not harmful.

As stated by Jennings (1987):

There is a tremendous sense of urgency and alarm in the treatment of intimate partner violence—and rightly so. After all, protecting the physical and emotional safety of women and their children is the first priority. Consequently, clinicians feel a primary obligation to “do something” immediately and decisively to halt and prevent violence (p. 204).

But as the above review has indicated, doing something may not help. As McCord (2003) so wisely noted, “Unless social programs are evaluated for potential harm as well as benefit, safety as well as efficacy, the choice of which social programs to use will remain a dangerous guess” (p. 16). It is clear that we need to be guided by rigorous research in helping us set our course. While better research is needed to determine the effectiveness of court‐mandated BIPs, the results from the meta‐analysis do not provide confidence that these programs will be found to be effective. Therefore, it would prove beneficial for the criminal justice system to begin looking at other types of interventions for addressing the problem of intimate partner violence. But these interventions must be tied to rigorous evaluations to determine their full impact. In other words, we recommend the use of pilot studies joined to an experimental design, as was suggested almost 20 years ago by Berk et al. (1985), as the preferred path for finding effective programs that can meet the challenge that intimate partner violence presents. Such a course of action would be especially prudent in these times of limited resources. More than that, victims and taxpayers are deserving of such evidence‐based decision‐making.

Unfortunately, what we are suggesting is not possible in many jurisdictions today in that their statutes now require that, upon conviction for intimate partner violence, individuals must be mandated into a BIP, not atypically based upon the Duluth Model (Babcock & Taillade, 2000). The end result is that judges, prosecutors and probation officers continue to send batterers to these treatment programs even as they have grave doubts about their effectiveness. Alternate programs cannot be implemented and tested even as evidence builds indicating that BIPs, at least as designed and implemented in the studies reviewed here, may not be effective.

### 7.2 Implications for research

The research implication growing out of this synthesis is that additional experiments need to be conducted to more clearly decipher the effectiveness of court‐mandated BIPs, focusing on new variants of these programs. If we are to test the ability of courts to mandate change, these future experiments must ensure samples of batterers who are representative of the larger convicted batterer population rather than a smaller subset of selected batterers. Additionally, these studies must attend to the importance of maintaining high victim retention so as to better ascertain any positive or negative effects from this mandated intervention. Finally, additional research is needed to better understand the validity and reliability of official report and victim report measures used in these studies and how they might be affected by treatment assignment. We would also encourage research into truly innovative approaches to addressing this problem that are radically distinct from the existing programs.

## ROLES AND RESPONSIBILITIES

Lynette Feder and David Wilson conducted the initial with search assistance from Sabrina Austin. Lynette Feder supervised Tamara Dimitrijevska‐Markoski in updating the search, with support from Ajima Olaghere. David Wilson and Ajima Olaghere coded the new studies, performed the updated analyses and revised the report.

## SOURCES OF SUPPORT

The Smith‐Richardson Foundation provided support for the original review.

## DECLARATIONS OF INTEREST

Lynette Feder was the primary investigator of an experiment assessing the effectiveness of a court‐mandated counseling program conducted in Broward County, Florida. To best counter the potential conflict of interest, the review was made as transparent as possible and included collaborators who had not been involved in any of the prior research reviewed here and who handled the coding for that study. There are no other none potential conflicts of interest among the authors.

## Supporting information

Supporting information

## Footnotes

1 No longer available or maintained.

## References

[cl21151-bib-0154] Blatch, C. , O'Sullivan, K. , Delaney, J. J. , van Doorn, G. , & Sweller, T. (2016). Evaluation of an Australian domestic abuse program for offending males. Journal of Aggression, Conflict and Peace Research, 8(1), 4–20.

[cl21151-bib-0001] Chen, H. , Bersani, C. , Myers, S. , & Denton, R. (1989). Evaluating the effectiveness of a court sponsored treatment program. Journal of Family Violence, 4, 309–322.

[cl21151-bib-0002] Davis, R. C. , Taylor, B. G. , & Maxwell, C. D. (2000). Does batterer treatment reduce violence? A randomized experiment in Brooklyn. National Institute of Justice.

[cl21151-bib-0003] Dunford, F. W. (2000). The San Diego Navy experiment: An assessment of interventions for men who assault their wives. Journal of Consulting and Clinical Psychology, 68, 468–476. 10883563 10.1037//0022-006x.68.3.468

[cl21151-bib-0004] Dutton, D. (1986). The outcome of court‐mandated treatment for wife assault: A quasi‐experimental evaluation. Violence and Victims, 1(3), 163–175. 3154147

[cl21151-bib-0005] Feder, L. , & Forde, D. (2000). *A test of the efficacy of court‐mandated counseling for domestic violence offenders: The Broward experiment* (Final report, Grant NIJ‐96WT‐NX‐0008). Washington, DC: National Institute of Justice.

[cl21151-bib-0006] Feder, L. , & Dugan, L. (2002). A test of the efficacy of court mandated counseling for domestic violence offenders: The Broward Experiment. Justice Quarterly, 19(2), 343–375.

[cl21151-bib-0149] Ferrer‐Perez, V. A. , & Bosch‐Fiol, E. (2018). Batterer intervention programs in Spain: An analysis of their effectiveness. International Journal of Offender Therapy and Comparative Criminology, 62(4), 885–897. 27707933 10.1177/0306624X16672455

[cl21151-bib-0007] Gordon, J. A. , & Moriarty, L. J. (2003). The effects of domestic violence batterer treatment on domestic violence batterer treatment on domestic violence recidivism. Criminal Justice and Behavior, 30(1), 118–134.

[cl21151-bib-0008] Harrell, A. (1991). Evaluation of court‐ordered treatment for domestic violence offenders (Final report). National Institute of Justice.

[cl21151-bib-0009] Jones, A. S. , & Gondolf, E. W. (2002). Assessing the effect of batterer program completion on re‐assault: An instrumental variables analysis. Journal of Quantitative Criminology, 18(1), 71–98.

[cl21151-bib-0150] Labriola, M. , Rempel, M. , & Davis, R. C. (2002). *Testing the effectiveness of batterer programs and judicial monitoring: Results from a randomized trial at the Bronx misdemeanor domestic violence court (Final report submitted to the National Institute of Justice).* Center for Court Innovation.

[cl21151-bib-0151] Miller, S. (2010). Discussing the Duluth curriculum: Creating a process of change for men who batter. Violence Against Women, 16(9), 1007–1021. 20710001 10.1177/1077801210379318

[cl21151-bib-0010] Palmer, S. , Brown, R. , & Barrera, M. (1992). Group treatment program for abusive husbands: Long‐term evaluation. American Journal of Orthopsychiatry, 62(2), 276283. 10.1037/h0079336 1580345

[cl21151-bib-0152] Pence, E. , Paymar, M. , & Ritmeester, T. (1993). *Education groups for men who batter: The Duluth model*. Springer.

[cl21151-bib-0011] Syers, M. , & Edleson, J. (1992). The combined effects of coordinated criminal justice intervention in woman abuse. Journal of Interpersonal Violence, 7, 490–502.

[cl21151-bib-0153] United States Attorney General's Task Force on Family Violence & United States, Department of Justice. (1984). Attorney General's task force on family violence. Washington, DC: US Department of Justice.

[cl21151-bib-0012] Alaska Judicial Council (1999). Evaluation of pilot program for misdemeanor domestic violence offenders (Final Report).

[cl21151-bib-0013] American Medical Association (1995). A coordinated approach to reducing family violence: conference highlights.

[cl21151-bib-0014] Aubertin, N. , & Laporte, P. (1999). Contrecoups: A program of therapy for spousal and family violence. Forum on Corrections Research, 11(1), 3–5.

[cl21151-bib-0015] Babcock, J. , & Steiner, R. (1999). The relationship between treatment, incarceration, and recidivism of battering: A program evaluation of Seattle's coordinated community response to domestic violence. Journal of Family Psychology, 13(1), 46–59.

[cl21151-bib-0016] Barrera, M. E. , Palmer, S. E. , Brown, R. A. , & Kalher, S. (1994). Characteristics of court involved and non‐court‐involved men who abuse their wives. Journal of Family Violence, 9(4), 333–345.

[cl21151-bib-0017] Dobash, R. , Dobash, R. , Cavanagh, K. , & Lewis, R. (1996a). Re‐education programmes for violent men: An evaluation. HMSO.

[cl21151-bib-0018] Dobash, R. , Dobash, R. , Cavanagh, K. , & Lewis, R. (1996b). Research evaluation of programmes for violent men. In R. E. Dobash , R. P. Dobash , K. Cavanagh & R. Lewis (Eds.), Changing violent men. Sage Publications.

[cl21151-bib-0019] Dobash, R. P. , Dobash, R. E. , Cavanaugh, K. , & Lewis, R. (1999). A research evaluation of British programmes for violent men. Journal of Social Policy, 28(2), 205–233.

[cl21151-bib-0020] Dobash, R. E. , & Dobash, R. P. (2000). Evaluating criminal justice interventions for domestic violence. Crime and Delinquency, 46(2), 252–270.

[cl21151-bib-0021] Dobash, R. P. , Dobash, R. E. , Cavanagh, K. , & Lewis, R. (2000). Confronting violent men. In J. Hanmer & C. Itzin (Eds.), Home truths about domestic violence: Feminist influences on policy and practice a reader (pp. 289–309). Routledge.

[cl21151-bib-0022] Dutton, D. , Bodnarchuk, M. , Kropp, R. , Hart, S. , & Ogloff, J. (1997). Wife assault treatment and criminal recidivism: An 11 year follow‐up. International Journal of Offender Therapy and Comparative Criminology, 41(1), 9–23.

[cl21151-bib-0023] Edleson, J. L. , & Grusznski, R. J. (1988). Treating men who batter: Four years of outcome data from the Domestic Abuse Project. Journal of Social Science Research, 12(1‐2), 3–22.

[cl21151-bib-0024] Flournoy, P. (1993). A comparison of groups for men who batter. (Doctoral dissertation, Washington State University) Dissertation Abstracts International: Section B: The Sciences & Engineering, 61(9‐B), 4989.

[cl21151-bib-0025] Ford, D. A. (1991). Preventing and provoking wife battery through criminal sanctioning: A look at the risks. In D. D. Knudsen & J. L. Miller (Eds.), Abused and battered: Social and legal responses of family violence. Social Institutions and social change (pp. 191–209). Aldine de Gruyter.

[cl21151-bib-0026] Ford, D. , & Regoli, M. (1992). The preventative impacts of policies for prosecuting wife batterers. In E. S. Buzawa & C. G. Buzawa (Eds.), Domestic violence: The changing CJ response (pp. 181–208). Auburn House.

[cl21151-bib-0027] Ford, D. , & Regoli, M. (1993). The Indianapolis domestic violence prosecution experiment (Final Report), Rockville, MD: National Institute of Mental Health. (NIMH #15161‐13).

[cl21151-bib-0028] Ford, D. , & M. (1993). The criminal prosecution of wife assaulters: Process, problems & effects. In Z. Hilton (Ed.), Legal responses to wife assault: Current trends and evaluation. Sage Publications.

[cl21151-bib-0029] Gamache, D. , Edleson, J. , & Schock, M. (1988). Coordinated police, judicial, and social service response to woman battering: A multiple‐baseline evaluation across three communities. In G. Hotaling , D. Finkelhor , J. T. Kirkpatrick & M. Straus (Eds.), Coping with family violence: Research and policy perspectives (pp. 193–209). Sage Publications.

[cl21151-bib-0030] Goldkamp, J. , Weiland, D. , Collins, M. , & White, M. (1996). The role of drug and alcohol abuse in domestic violence and its treatment: Dade county's domestic violence court experiment (Final Report), Washington, DC: National Institute of Justice (NCJRS #163410).

[cl21151-bib-0031] Gondolf, E. W. , & Jones, A. S. (2001). The program effect of batterer programs in three cities. Violence and Victims, 16(6), 693–704. 11863066

[cl21151-bib-0032] Gondolf, E. W. (2000). A 30‐month follow‐up of court referred batterers in four cities. International Journal of Offender Therapy & Comparative Criminology, 44(1), 111128.

[cl21151-bib-0033] Gondolf, E. W. (1999). A comparison of four batterer intervention systems: Do court referral, program length, and services matter? Journal of Interpersonal Violence, 14(1), 41–61.Jan.

[cl21151-bib-0034] Gondolf, E. W. (1988). How some men stop their abuse: An explanatory programs evaluation. In G. T. Hotaling , D. Finkelhor , J. T. Kirkpatrick & M. A. Straus (Eds.), Coping with family violence: Research and policy perspectives (pp. 129–144). Sage Publications.

[cl21151-bib-0035] Gondolf, E. W. (1997). Patterns of re‐assault in batterer programs. Violence and Victims, 12(4), 373–387. 9591355

[cl21151-bib-0036] Hamberger, K. , & Hastings, J. (1988). Skills training for treatment of spouse abusers: An outcome study. Journal of Family Violence, 3(2), 121–1130.

[cl21151-bib-0037] Hamm, M. , & Kite, J. (1991). The role of offender rehabilitation in family violence policy: The Batterers Anonymous Experiment. Criminal Justice Review, 16(2), 227–248.

[cl21151-bib-0038] Heckert, D. A. , & Gondolf, E. W. (2000). The effect of perceptions of sanctions on batterer program outcomes. Journal of Research in Crime and Delinquency, 37(4), 369–391.

[cl21151-bib-0046] Healey, K. , & Smith, C. (1998). Batterer programs: What criminal justice agencies need to know. U.S. Department of Justice.

[cl21151-bib-0039] Jolin, A. , Feyerherm, W. , Fountain, R. , & Friedman, S. (1998). Beyond arrest: The Portland, Oregon domestic violence experiment (Final Report), Washington, DC: U.S. Department of Justice (NCJRS #179968).

[cl21151-bib-0040] Kistenmacher, B. R. (2001). Motivational Interviewing as a mechanism for change in men who batter: a randomized controlled trial (Doctoral dissertation, University of Oregon)Dissertation Abstracts International: Section B: The Sciences & Engineering, 61(9‐B), 4989.

[cl21151-bib-0041] Krmpotich, S. , & Eckberg, D. (2000). Domestic assault program evaluation: Final (2year) results, Minneapolis: Hennepin County Department of Community Corrections.

[cl21151-bib-0042] Ley, D. J. (2001). Effectiveness of a court‐ordered domestic violence treatment program: A clinical utility study (Doctoral dissertation, University of New Mexico). Dissertation Abstracts International: Section B: The Sciences & Engineering, 62(4B), 2056.

[cl21151-bib-0043] Morrell, T. , Elliott, J. , Murphy, C. , & Taft, C. (2003). Cognitive behavioral and supportive group treatment for partner‐violent men. Behavior Therapy, 34, 77–95.

[cl21151-bib-0044] Murphy, C. M. , Musser, P. H. , & Maton, K. I. (1998). Coordinated community intervention for domestic abusers: Intervention system involvement and criminal recidivism. Journal of Family Violence, 13(3), 263–284.

[cl21151-bib-0045] National Institute of Justice (1998). Legal interventions in family violence: Research findings and policy implications. U.S. Department of Justice.

[cl21151-bib-0047] Newell, R. G. (1994). The effectiveness of court‐mandated counseling for domestic violence: An outcome study (Doctoral dissertation, University of Toledo. Dissertation Abstracts International: Section A: The Sciences & Engineering, 55(05), 1193.

[cl21151-bib-0048] Pellegrini, K. L. (1999). Analysis of a violence intervention program: Population, treatment compliance, and recidivism (Doctoral dissertation, George Fox University) Dissertation Abstracts International: Section B: The Sciences & Engineering, 60(10‐B), 5231.

[cl21151-bib-0049] Petrik, N. , Gildersleeve‐High, L. , & McEllistrem, J. (1994). The reduction of male abusiveness as a result of treatment: Reality or myth? Journal of Family Violence, 9, 307–316.

[cl21151-bib-0050] Petrucci, C. J. (2002). A qualitative & quantitative analysis of a specialized DV court that utilizes therapeutic jurisprudence (Doctoral dissertation, University of California, Los Angeles).

[cl21151-bib-0051] Taft, C. , Murphy, C. , Elliot, J. , & Morrell, T. (2001). Attendance‐enhancing procedures in group counseling for domestic abuse. Journal of Consulting Psychology, 48(1), 5160.

[cl21151-bib-0052] Taylor, B. , Davis, R. , & Maxwell, C. (2001). The effects of a group batterer treatment program: A randomized experiment in Brooklyn. Justice Quarterly, 18(1), 171–201.

[cl21151-bib-0053] Tolman, R. M. , & Bhosley, G. (1991). The outcome of participation in a shelter‐sponsored program for men who batter. In D. D. Knudsen & J. L. Miller (Eds.), Abused and Battered: Social and legal responses of family violence. Social Institutions and social change (pp. 191–209). Aldene de Gruyter.

[cl21151-bib-0054] Tutty, L. M. , Bidgood, B. A. , Rothery, M. A. , & Bidgood, P. (2001). An evaluation of men's batterer treatment groups. Research on Social Work Practice, 11(6), 645–670.

[cl21151-bib-0055] Waldo, M. (1988). Relationship enhancement counseling groups for wife abusers. Journal of Mental Health Counseling, 10(1), 37–45.

[cl21151-bib-0056] Adams, D. , & McCormick, A. (1982). Men unlearning violence: A group approach based on the collective model. In M. Roy (Ed.), The abusive partner: An analysis of domestic battering (pp. 170–197). Van Nostrand Reinhold.

[cl21151-bib-0057] Alexander, P. C. , Morris, E. , Tracy, A. , & Frye, A. (2010). Stages of change and the group treatment of batterers: A randomized clinical trial. Violence and Victims, 25(5), 571–587. 21061865 10.1891/0886-6708.25.5.571

[cl21151-bib-0058] Arce, R. , Arias, E. , Novo, M. , & Fariña, F. (2020). Are interventions with batterers effective? A meta‐analytical review. Psychosocial Intervention, 29(3), 153–164.

[cl21151-bib-0059] Arias, E. , Arce, R. , & Vilariño, M. (2013). Batterer intervention programmes: A meta‐analytic review of effectiveness. Psychosocial Intervention, 22(2), 153–160.

[cl21151-bib-0060] Babcock, J. C. , & Taillade, J. (2000). Evaluating interventions for men who batter. In J. Vincent & E. Jouriles (Eds.), Domestic violence: Guidelines for research‐informed practice (pp. 37–77). Jessica Kingsley.

[cl21151-bib-0061] Babcock, J. C. , Green, C. E. , & Robie, C. (2004). Does batterers' treatment work? A meta‐analytic review of domestic violence treatment. Clinical Psychology Review, 23(8), 1023–1053. 14729422 10.1016/j.cpr.2002.07.001

[cl21151-bib-0062] Babcock, J. , Armenti, N. , Cannon, C. , Lauve‐Moon, K. , Buttell, F. , Ferreira, R. , Cantos, A. , Hamel, J. , Kelly, D. , Jordan, C. , Lehmann, P. , Leisring, P. A. , Murphy, C. , O'Leary, K. D. , Bannon, S. , Salis, K. L. , & Solano, I. (2016). Domestic violence perpetrator programs: A proposal for evidence‐based standards in the United States. Partner Abuse, 7(4), 356–460.

[cl21151-bib-0063] Banks, J. , Kini, S. , & Babcock, J. (2013). Interventions that work to stop intimate partner violence. In L. A. Craig , L. Dixon & T. A. Gannon (Eds.), What works in offender rehabilitation: An evidence‐based approach to assessment and treatment (pp. 159–172). Wiley. 10.1002/9781118320655.ch9

[cl21151-bib-0064] Berk, R. , Campbell, A. , Klap, R. , & Western, B. (1992). The deterrent effect of arrest in incidents of domestic violence: A Bayesian analysis of four field experiments. American Sociological Review, 57(5), 698–708.

[cl21151-bib-0065] Berk, R. , Boruch, T. , Chambers, F. , Rossi, P. , & Witte, S. (1985). Social policy experimentation: A position paper. Evaluation Review, 9(4), 387–429.

[cl21151-bib-0066] Black, M. C. , Basile, K. C. , Breiding, M. J. , Smith, S. G. , Walters, M. L. , Merrick, M. T. , Chen, J. , & Stevens, M. R. (2011). *The National Intimate Partner and Sexual Violence Survey (NISVS): 2010 Summary Report*. Atlanta, GA: National Center for Injury Prevention and Control, Centers for Disease Control and Prevention.

[cl21151-bib-0067] Brisson, N. (1981). Battering husbands: A survey of abusive men. Victimology, 6, 338344.

[cl21151-bib-0068] Buzawa, E. S. , & Buzawa, C. G. (2017). The evolution of the response to domestic violence in the United States. In E. Buzawa & C. Buzawa (Eds.), Global responses to domestic violence. Springer. 10.1007/978-3-319-56721-1_4

[cl21151-bib-0069] Cannon, C. , Hamel, J. , Buttell, F. , & Ferreira, R. J. (2016). A survey of domestic violence perpetrator programs in the United States and Canada: Findings and implications for policy and intervention. Partner abuse, 7(3), 226–276.

[cl21151-bib-0070] Catalano, S. , Smith, E. , Snyder, H. , & Rand, M. (2009). Female victims of violence. US Department of Justice.

[cl21151-bib-0071] Chalk, R. , & King, P. (1998). Violence in families: Assessing prevention and treatment programs. National Academy Press.

[cl21151-bib-0072] Cheng, S. Y. , Davis, M. , Jonson‐Reid, M. , & Yaeger, L. (2019). Compared to what? A meta‐analysis of batterer intervention studies using nontreated controls or comparisons. Trauma, Violence & Abuse, 1–16. 10.1177/1524838019865927 31359840

[cl21151-bib-0073] Cluss, P. , & Bodea, A. (2011). *The effectiveness of batterer intervention programs: A literature review and recommendations for next steps* (Unpublished manuscript). University of Pittsburgh, Pittsburgh, PA.

[cl21151-bib-0074] Davis, R. , & Taylor, B. (1999). Does batterer treatment reduce violence? Women & Criminal Justice, 10, 69–93.

[cl21151-bib-0075] Davis, R. , Maxwell, C. , & Taylor, B. (2003). The Brooklyn experiment. In S. Jackson , L. Feder , D. Forde , R. Davis , C. Maxwell & B. Taylor (Eds.), Batterer intervention programs: Where do we go from here? (pp. 15–21). Department of Justice.

[cl21151-bib-0076] Desmarais, S. L. , Reeves, K. A. , Nicholls, T. L. , Telford, R. P. , & Fiebert, M. S. (2012). Prevalence of physical violence in intimate relationships, Part 1: Rates of male and female victimization. Partner Abuse, 3(2), 140–169.

[cl21151-bib-0077] Dutton, D. (1984). Interventions into the problem of wife assault: Therapeutic, policy and research implications. Canadian Journal of Behavioral Science, 16(4), 281–297.

[cl21151-bib-0078] Dutton, D. (1988). Research advances in the study of wife assault: Etiology and prevention. Law and Mental Health, 4, 161–220.

[cl21151-bib-0079] Dutton, D. , & McGregor, B. (1991). The symbiosis of arrest and treatment for wife assault: The case for combined intervention. In M. Steinman (Ed.), Woman battering: Policy responses (pp. 131–154). Anderson Publishing Company.

[cl21151-bib-0080] Eckhardt, C. I. , & Utschig, A. C. (2007). Assessing readiness to change among perpetrators of intimate partner violence: Analysis of two self‐report measures. Journal of Family Violence, 22(5), 319–330.

[cl21151-bib-0081] Eckhardt, C. I. , Murphy, C. M. , Whitaker, D. J. , Sprunger, J. , Dykstra, R. , & Woodard, K. (2013). The effectiveness of intervention programs for perpetrators and victims of intimate partner violence. Partner Abuse, 4(2), 196–231.

[cl21151-bib-0082] Edleson, J. , & Brygger, M. (1995). Gender differences in reporting of battering incidences. In S. Stith & M. Straus (Eds.), Understanding partner violence: Prevalence, causes, consequences and solutions (pp. 45–50). National Council of Family Relations.

[cl21151-bib-0083] Eisikovits, Z. , & Edleson, J. (1989). Intervening with men who batter: A critical review of the literature. Social Service Review, 63, 384–414.

[cl21151-bib-0084] Farley, D. , & Magill, J. (1988). An evaluation of a group program for men who batter. Social Work with Groups, 11(3), 53–65.

[cl21151-bib-0085] Feazell, C. , Mayers, R. , & Deschner, J. (1984). Services for men who batter: Implications for programs and policies. Family Relations, 33, 217–223.

[cl21151-bib-0086] Feder, L. (1997). Domestic violence and police response in a pro‐arrest jurisdiction. Women and Criminal Justice, 8(4), 79–98.

[cl21151-bib-0087] Feder, L. , & Wilson, D. B. (2006). Protocol: Court‐mandated interventions for individuals convicted of domestic violence: A Campbell Collaboration Systematic Review. Campbell Systematic Reviews, 2(1), 1–31. 10.1002/cl2.1151 PMC8356297 37133255

[cl21151-bib-0088] Feder, L. , Wilson, D. B. , & Austin, S. (2008). Court‐mandated interventions for individuals convicted of domestic violence. Campbell Systematic Reviews, 4(1), 1–46. 10.1002/cl2.1151 PMC8356297 37133255

[cl21151-bib-0089] Follingstad, D. R. , & Rogers, M. J. (2013). Validity concerns in the measurement of women's and men's report of intimate partner violence. Sex Roles, 69(3‐4), 149–167.

[cl21151-bib-0090] Ford, D. , & Regoli, M. J. (1993). The criminal prosecution of wife assaulters. In Z. Hilton (Ed.), Legal responses to wife assault: Current trends and evaluation (pp. 127–164). Sage.

[cl21151-bib-0091] Freeman, A. J. , Schumacher, J. A. , & Coffey, S. F. (2015). Social desirability and partner agreement of men's reporting of intimate partner violence in substance abuse treatment settings. Journal of Interpersonal Violence, 30(4), 565–579. 24923888 10.1177/0886260514535263 PMC8336652

[cl21151-bib-0092] Gondolf, E. (1987). Seeing through smoke and mirrors: A guide to batterer program evaluations. Response, 10, 16–19.

[cl21151-bib-0093] Gondolf, E. (1987). Evaluating programs for men who batter: Problems and prospects. Journal of Family Violence, 2(1), 95–108.

[cl21151-bib-0094] Gondolf, E. (1998). Do batterer programs work? A 15 month follow‐up of multi‐site evaluation. Domestic Violence Report, 3(5), 65–80.

[cl21151-bib-0095] Goolkasian, G. (1986). Confronting domestic violence: The role of criminal court judges. National Institute of Justice.

[cl21151-bib-0096] Gover, A. R. (2017). A review of state standards for batterer intervention treatment programs and the Colorado Model. Court Review, 53, 36–41.

[cl21151-bib-0097] Hamberger, L. K. , & Hastings, J. (1989). Counseling male spouse abusers: Characteristics of treatment completers and dropouts. Violence and Victims, 4(1), 275–286. 2487139

[cl21151-bib-0098] Hamberger, L. K. , & Hastings, J. (1993). Court‐mandated treatment of men who assault their partner. In Z. Hilton (Ed.), Legal responses to wife assault: Current trends and evaluation (pp. 188–229). Sage.

[cl21151-bib-0099] Hamilton, L. , Koehler, J. A. , & Lösel, F. A. (2012). Domestic violence perpetrator programs in Europe, part I: A survey of current practice. International Journal of Offender Therapy and Comparative Criminology, 57(10), 1189–1205. 23267241 10.1177/0306624X12469506

[cl21151-bib-0100] Hasselblad, V. , & Hedges, L. V. (1995). Meta‐analysis of screening and diagnostic tests. Psychological Bulletin, 117, 167–178. 7870860 10.1037/0033-2909.117.1.167

[cl21151-bib-0101] Healey, K. , Smith, C. , & O'Sullivan, C. (1998). Batterer intervention: Program approaches and criminal justice strategies. Department of Justice.

[cl21151-bib-0102] Healey, K. , & Smith, C. (1998). Batterer programs: What criminal justice agencies need to know. National Institute of Justice.

[cl21151-bib-0103] Hedges, L. V. , Tipton, E. , & Johnson, M. C. (2010). Robust variance estimation in meta‐regression with dependent effect size estimates. Research Synthesis Methods, 1(1), 39–65. 26056092 10.1002/jrsm.5

[cl21151-bib-0104] Helfritz, L. E. , Stanford, M. S. , Conklin, S. M. , Greve, K. W. , Villemarette‐Pittman, N. R. , & Houston, R. J. (2006). Usefulness of self‐report instruments in assessing men accused of domestic violence. The Psychological Record, 56(2), 171–180.

[cl21151-bib-0105] Hilberman, E. (1980). Overview: The “wife‐beater's wife” reconsidered. American Journal of Psychiatry, 137(11), 1336–1347. 7435666 10.1176/ajp.137.11.1336

[cl21151-bib-0106] Hirschel, J. D. , & Hutchinson, I. (1992). Female spouse abuse and the police response: The Charlotte, North Carolina Experiment. Journal of Criminal Law and Criminology, 83(1), 73–119.

[cl21151-bib-0107] Hotaling, G. , & Sugarman, D. (1986). An analysis of risk markers in husband to wife violence: The current state of knowledge. Violence and Victims, 1(2), 101–124. 3154143

[cl21151-bib-0108] Jennings, J. (1987). History and issues in the treatment of battering men: A case for unstructured group therapy. Journal of Family Violence, 2(3), 193–213.

[cl21151-bib-0109] Johnson, J. , & Kanzler, D. (1993). Treating domestic violence: Evaluating the effectiveness of a domestic violence diversion program. Studies in Symbolic Interaction, 15, 271–289.

[cl21151-bib-0110] Krahé, B. (2018). Violence against women. Current Opinion in Psychology, 19, 6–10. 29279224 10.1016/j.copsyc.2017.03.017

[cl21151-bib-0111] Langan, P. , & Innes, C. (1986). Preventing domestic violence against women. National Institute of Justice.

[cl21151-bib-0112] Maxwell, C. D. , Davis, R. C. , & Taylor, B. G. (2010). The impact of length of domestic violence treatment on the patterns of subsequent intimate partner violence. Journal of Experimental Criminology, 6(4), 475–497.

[cl21151-bib-0113] Mazerolle, L. , Eggins, E. , Sydes, M. , Hine, L. , McEwan, J. , Norrie, G. , & Somerville, A. (2018). Criminal justice responses to domestic and family violence: A rapid review of the literature. University of Queensland.

[cl21151-bib-0114] McCord, J. (2003). Cures that harm: Unanticipated outcomes of crime prevention programs. Annals of the American Academy of Political and Social Science, 587, 1630.

[cl21151-bib-0115] McDonald, R. , Jouriles, E. , Ranisetty‐Mikler, S. , Caetano, R. , & Green, C. (2006). Estimating the number of American children living in partner‐violent families. Journal of Family Psychology, 20(1), 137–142. 16569098 10.1037/0893-3200.20.1.137

[cl21151-bib-0116] Miller, T. , Cohen, M. , & Wiersema, B. (1996). Victim costs and consequences: A new look. National Institute of Justice.

[cl21151-bib-0117] Morgan, R. E. , & Oudekerk, B. A. (2019). Criminal victimization, 2018. Bureau of Justice Statistics, U.S. Department of Justice Office of Justice Programs. https://www.bjs.gov/content/pub/pdf/cv18.pdf

[cl21151-bib-0118] Myhill, A. , & Johnson, K. (2016). Police use of discretion in response to domestic violence. Criminology & Criminal Justice, 16(1), 3–20.

[cl21151-bib-0119] Murphy, C. M. , & Ting, L. A. (2010). Interventions for perpetrators of intimate partner violence: A review of efficacy research and trends. Partner Abuse, 1, 26–44.

[cl21151-bib-0120] Pence, E. (1983). The Duluth domestic abuse intervention project. Hamline Law Review, 6, 247–275.

[cl21151-bib-0121] Policastro, C. , & Payne, B. K. (2013). The blameworthy victim: Domestic violence myths and the criminalization of victimhood. Journal of Aggression, Maltreatment & Trauma, 22(4), 329–347.

[cl21151-bib-0122] Pirog‐Good, M. , & Stets‐Kealey, J. (1985). Male batterers and battering prevention programs: A national survey. Response, 8, 8–12.

[cl21151-bib-0123] Price, B. J. , & Rosenbaum, A. (2009). Batterer intervention programs: A report from the field. Violence and Victims, 24(6), 757–770. 10.1891/0886-6708.24.6.757 20055213

[cl21151-bib-0124] Roberts, A. (1982). A national survey of services for batterers. In M. Roy (Ed.), The abusive partner: An analysis of domestic battering (pp. 230–243). Van Nostrand Reinhold.

[cl21151-bib-0125] Rosenfeld, B. (1992). Court‐ordered treatment of spouse abuse. Clinical Psychology Review, 12, 205–226.

[cl21151-bib-0126] Saltzman, L. E. , Fanslow, J. L. , McMahon, P. M. , & Shelley, G. A. (1999) *Intimate partner violence, surveillance: Uniform definitions and recommended data elements, version 1.0*. National Center for Injury Prevention and Control, Atlanta GA.

[cl21151-bib-0127] Santirso, F. A. , Gilchrist, G. , Lila, M. , & Gracia, E. (2020). Motivational strategies in interventions for intimate partner violence offenders: A systematic review and meta‐analysis of randomized controlled trials. Psychosocial Intervention, 29(3), 175–190.

[cl21151-bib-0128] Saunders, D. (1996). Interventions for men who batter: Do we know what works? In Session: Psychotherapy in Practice, 2(3), 81–93.

[cl21151-bib-0129] Shadish, W. R. , Cook, T. D. , & Campbell, D. T. (2002). Experimental and quasi‐experimental designs for generalized causal inference. Houghton Mifflin.

[cl21151-bib-0130] Sherman, L. (1992). The influence of criminology on criminal law: Evaluating arrests for misdemeanor domestic violence. Journal of Criminal Law and Criminology, 83, 1–45.

[cl21151-bib-0131] Smedslund, G. , Dalsbø, T. K. , Steiro, A. K. , Winsvold, A. , & Clench‐Aas, J. (2011). Cognitive behavioural therapy for men who physically abuse their female partner. Campbell Systematic Reviews, 7(1), 1–25. 10.1002/14651858.CD006048.pub2 PMC12047670 17636823

[cl21151-bib-0132] Smith, S. , Zhang, X. , Basile, K. , Merrick, M. , Wang, J. , Kresnow, M. , & Chen, J. (2018). *The national intimate partner and sexual violence survey: 2015 data brief—Updated release*. Atlanta, GA: National Center for Injury Prevention and Control, Centers for Disease Control and Prevention.

[cl21151-bib-0133] Snyder, D. , & Scheer, N. (1981). Predicting disposition following brief residence at a shelter for battered women. American Journal of Community Psychology, 9, 559–566.

[cl21151-bib-0134] Soleymani, S. , Britt, E. , & Wallace‐Bell, M. (2018). Motivational interviewing for enhancing engagement in intimate partner violence (IPV) treatment: A review of the literature. Aggression and Violent Behavior, 40, 119–127.

[cl21151-bib-0135] Sonkin, D. J. (1988). The male batterer: Clinical and research issues. Violence and Victims, 3(1), 65–79. 3154173

[cl21151-bib-0136] Stover, C. S. , Meadows, A. L. , & Kaufman, J. (2009). Interventions for intimate partner violence: Review and implications for evidence‐based practice. Professional Psychology: Research and Practice, 40(3), 223–233.

[cl21151-bib-0137] Straus, M. (1991). Conceptualization and measurement of battering: Implications for public policy. In M. Steinman (Ed.), Woman battering: Policy responses (pp. 19–47). Anderson.

[cl21151-bib-0138] Straus, M. , Hamby, S. , Boney‐McCoy, S. , & Sugarman, D. (1996). The revised Conflict Tactics Scale (CTS2): Development and preliminary psychometric data. Journal of Family Issues, 17(3), 283–316.

[cl21151-bib-0139] Sullivan, C. , Rumptz, M. , Campbell, R. , Eby, K. , & Davidson, W. (1996). Retaining participants in longitudinal community research: A comprehensive protocol. Journal of Applied Behavioral Science, 32(3), 262–276.

[cl21151-bib-0140] Taylor, B. , Davis, R. , & Maxwell, C. (2001). The effects of a group batterer treatment program: A randomized experiment in Brooklyn. Justice Quarterly, 18(1), 171–201.

[cl21151-bib-0141] Tjaden, P. , & Thoennes, N. (2000). Prevalence and consequences of male‐to‐female and female‐to‐male intimate partner violence as measured by the National Violence Against Women Survey. Violence Against Women, 6(2), 142–161.

[cl21151-bib-0142] Tolman, R. , & Bennett, L. (1990). A review of quantitative research on men who batter. Journal of Interpersonal Violence, 5, 87–118.

[cl21151-bib-0143] Tolman, R. , & Edleson, J. (1995). Intervention for men who batter: A review of research. In S. Stith & M. Straus (Eds.), Understanding partner violence: Prevalence, causes, consequences and solutions (pp. 262–273). National Council on Family Relations.

[cl21151-bib-0144] Viechtbauer, W. (2010). Conducting meta‐analyses in R with the metafor package. Journal of Statistical Software, 36(3), 1–48. http://www.jstatsoft.org/v36/i03/

[cl21151-bib-0145] Weisburd, D. , Lum, C. , & Petrosino, A. (2001). Does research design affect study outcomes in criminal justice? Annals of the American Academy of Political and Social Science, 578, 50–70.

[cl21151-bib-0146] Widom, C. S. (1992). The cycle of violence. US Department of Justice.

[cl21151-bib-0147] Zorza, J. (2003). New research: Broward County Experiment shows no benefit from batterer intervention programs. Domestic Violence Report, 8, 23–25.

