# Supplementary material for: Court‐mandated interventions for individuals convicted of domestic violence: An updated Campbell systematic review
Source: Campbell Syst Rev. 2021 Mar 14;17(1):e1151. doi: 10.1002/cl2.1151 (PMC8356297; doi:10.1002/cl2.1151)
Supplement: Supplementary file 1 — Supporting information [file CL2-17-e1151-s001.docx]

# Appendix A: Coding Forms

**Study Level Code Sheet**

Use one study level code sheet for each study.

**Identifying Information:**

1. Study (document) identifier StudyID ______

2. Cross reference document identifier CrossRef1 ______

3. Cross reference document identifier CrossRef2 ______

4. Cross reference document identifier CrossRef3 ______

5. Coder’s initials SCoder ______

6. Date coded SDate ___ - __ - __

**General Study Information:**

7. Author Author ________________________________

8. Funder (e.g., NIJ) Funder ________________________________

9. Geographical Location of Study SLocale ________________________________

10. Date range for participant entry into study (preferably when sample pulled):

StartDate ___ - __ - __

DoneDate: ___ - __ - __

11. Publication Type PubType ___

1. Book 2. Book Chapter

3. Journal (peer reviewed) 4. Federal Gov't Report

5. State/Local Gov't Report 6. Dissertation/Thesis

7. Unpublished (tech report, conference paper)

12. Number of treatment groups TxGrps ___

13. Number of control groups CgGrps ___

14. Is the same control group used in different contrasts

(1=yes, 0=no, 8=NA) SameCG ___

15. Is there any evidence of selective reporting of outcomes, such as only reporting outcomes with favorable results? (1 = Yes, 2 = Probably Yes, 3 = Probably No, 4 = No, 5 = No information) MissingDVs ___

**Treatment-Comparison Level Code Sheet**

Use one treatment-comparison level code sheet for each treatment-comparison within a study. For example, if a study has three treatment conditions and each is compared to a single control condition, code the information below separately for each treatment compared to the single control condition resulting in three treatment-comparison code sheets. Give each treatment-comparison a unique treatment-comparison identifier (TxID), such as 1, 2, 3, etc.

**Identifying Information:**

15. Study (document) identifier StudyID ______

16. Treatment-comparison identifier TxID ______

Label for this treatment __________________________________________

17. Coder's initials TCoder ______

**Nature of the Treatment:**

18. Type of treatment program (code all that apply, 1=yes, 0=no, 9=cannot tell):

(a) Cognitive-behavioral TxType1 ___

(b) Psychoeducational (including Duluth) TxType2 ___

(c) Feminist TxType3 ___

(d) Individual counseling TxType4 ___

(e) Marital counseling TxType5 ___

(f) Extensive Monitoring TxType6 ___

(g) Other ________________________________ TxType9 ___

19. Treatment existed in the community prior to the research study

(1=yes, 0=no, 9=cannot tell) TxExist ___

20. Treatment format (1=yes, 0=no, 9=cannot tell)

1. Group (men only) TxFrmat1 ___

2. Conjoint group TxFrmat2 ___

3. Individual (offender) TxFrmat3 ___

4. Individual (victim) TxFrmat4 ___

5. Conjoint (couples) individual TxFrmat5 ___

6. Other _________________________________ TxFrmat8 ___

21. Does the treatment group also receive probation?

(1=yes, 0=no, 9=cannot tell) TxProb ___

22. Average length of probation in weeks (4.3 weeks per month; 88=not applicable; 99=missing) TxProbLn ___

23. How voluntary is the offenders participation? TxVolun ___

1. Nonvoluntary (court mandated)

2. Court-mandated after agreement from offender

3. Completely voluntary

4. Some court-mandated, some voluntary

9. Cannot tell

24. If #4 above, specify the percent court mandated (888 if n/a) TxMand ______

25. Duration of the treatment program in weeks (99 if unknown) TxWeeks ___

26. Number of treatment sessions (99 if unknown, 88 if not applicable) TxNum ___

27. Mean number of sessions attended

(99 if unknown, 88 if not applicable) TxAttend _________

28. Length of a treatment session in hours (99 if unknown, 88 if not applicable)

TxHours1 ___

29. Total length of treatment in hours (99 if unknown, 88 if not applicable)

TxHours2 ___

30. Sanctions applied for failing to comply with treatment? TxSanc ___

1. Yes, typically

2. Yes, sporadically

3. No

9. Not indicated

31. How many sessions was considered successful completion of treatment?

(99 if unknown, 88 if not applicable) TxNumSuc ___

32. Percentage of treatment sample completing program, as defined by the researchers. (999 if missing) TxCompl ______

33. Evidence of adherence to the treatment protocol (i.e., fidelity of the treatment delivery separate from subject compliance; did the treatment providers adhere to the treatment model; any evidence of fidelity considered, including certification) TxFidel ___

1. Yes, evidence of treatment fidelity

2. Evidence of some deviation from treatment model

3. Evidence of serious deviation from treatment model

4. No mention of treatment fidelity

**Nature of the Control Condition:**

34. Who was included in the comparison group? CgVol ___

1. Voluntary treatment or program seekers only

2. Arrested individuals

3. Mix of the above individuals

35. What does the comparison group receive? (code 1=yes, 0=no, 9=not indicated for each)

1. No treatment or program CgType1 ___

2. Probation CgType2 ___

3. Jail or Prison CgType3 ___

4. Community service CgType4 ___

5. Some or no treatment (e.g., treatment no-show or drop out) CgType5 ___

6. Other _________________ CgType6 ___

7. Cannot tell CgType7 ___

36. Duration of comparison group program (in weeks, 99=missing): CgWeeks ___

37. Investigated compensation for control group

(1=yes, 0=no, 9=not indicated) CgComp ___

**Methodological Rigor:**

38. How were subjects assigned to conditions? TxRandom __

1. Random (simple)

2. Random (matching pairs)

1. Quasi-random (alternative cases, alternative blocks of cases)
2. Other ________________________________________

39. Missassignment rate (percentage of cases that violated the random assignment protocol) (999 if missing, 888 if not applicable)

(a) From treatment to control TxMsRte1 ______

(b) From control to treatment TxMsRte2 ______

(c) Total TxMsRte3 ______

40. How did the researchers handle violations to random

assignment? TxAnalyz ___

1. Analyzed as assigned

2. Analyzed as treated

3. Both 1 and 2 above (only code effect sizes for 1)

4. Removed cases

8. Not applicable

1. Not indicated

41. Type of quasi-experimental design (nature of comparison

group) CgNature ______

01. Historical comparison group

02. Judge or prosecutor did not court-mandate into treatment but gave them an alternative sanction (Specify: ____________________________________)

03. Treatment no-shows or drop-outs as the comparison group

04. Eligible domestic violence offenders from an alternative jurisdiction without a court mandated program

05. Matched samples comparison group (sample of non-court mandated domestic offenders drawn from a large pool in a manner designed to produce a

group with similar background characteristics to the court mandated group)

06. Domestic violence offenders referred but not accepted into the treatment program

07. Domestic violence offenders not arrested (e.g., Syers and Edleson study)

08. Other ____________________________________________

88. Not applicable (experimental design)

99. Not indicated

42. Who was included in the experimental group for the comparison? TxCg ___

1. All individuals assigned to treatment (includes rejects,

no-shows & drop-outs)

2. All individuals assigned to treatment (excludes rejects)

3. Only those who completed a specified amount of treatment

4. Only those who completed all treatment

8. Other ____________________________________________

43. Did the researchers test for baseline (pre-test) differences?

(1=yes; 0=no) TxDiff1 ___

44. If yes to above, nature of any pretest differences TxDiff2 ___

1. If n>100, no significant differences

2. If n<100, no substantive or significant differences

3. Minor differences or differences on variables unlikely to be

related to offending

1. Major or important difference
2. Not applicable

45. Baseline (pretest) differences judged to bias the results in

which direction? TxBias ___

1. Positive bias (treatment effect likely to be larger than it really is)

2. Negative bias (treatment effect likely to be smaller than it really is)

3. No bias (no differences or differences on variables that should

have no effect)

4. Cannot make a judgment (differences have an uncertain effect)

1. Cannot tell

45b. Risk of selection bias SelectBias ___

1. Low-risk

2. High-risk: Bias favors treatment

3. High-risk: Bias favors control

4. High-risk: Direction not predictable

5. Unclear-risk

46. Analysis of treatment effect statistically adjusted for baseline differences (e.g., logistics regression and ANCOVA that included baseline and background characteristics) (1=yes; 0=no; 9=cannot tell) StatCtrl ___

47. If not an experimental design, was matching used? (1=yes; 0=no; 8=na; 9=cannot tell)

MtchCtrl ___

48. Which, if any, of the following baseline/background characteristics were matched on or statistically controlled for in the estimate of the treatment effect? (1=yes; 0=no; 9=cannot tell)

1. Age Cov01 ___
2. Race/ethnicity Cov02 ___
3. Employment status Cov03 ___
4. Income Cov04 ___
5. Prior domestic violence history Cov05 ___
6. Prior violent history (general or nondomestic) Cov06 ___
7. Prior non-violent history Cov07 ___
8. Seriousness of present offense Cov08 ___
9. Education Cov09 ___
10. Marital status Cov10 ___
11. Alcohol or drug use Cov11 ___
12. Psychosocial or personality variables (e.g., MMPI, self-esteem) Cov12 ___

88. Other ________________________________________ Cov88 ___

**Sample Characteristics:**

Note: These questions apply to the sample characteristics for the subjects included in both the treatment and control conditions for this treatment-comparison contrast. If there are multiple treatment-comparison conditions and data are presented for the study as a whole, use the overall data.

49. Total sample size for this treatment comparison (at start of study)

(9999=missing) STotN _________

50. Treatment sample size (at start of study) (9999=missing) STxN _________

51. Control sample size (at start of study) (9999=missing) SCgN _________

52. Sample characteristics (1=yes, 0=no, 9=not indicated)

(a) nonvoluntary (court referred) SCtRef ___

(b) misdemeanor defendants/offenders SMisdem ___

(c) misdemeanor and felony defendants/offenders SMisFel ___

(d) voluntary SVol ___

(e) other ______________________________ SOther ___

53. Treatment sample's disposition (1=yes, 0=no, 9=not indicated)

(a) post-conviction TPostC ___

(b) conditional discharge TCDisch ___

(c) pretrial diversion (adjourned in contemplation of dismissal) TPrtrial ___

(d) military disposition TMiltary ___

(e) other ___________________________ TSOther ___

54. Control sample's disposition (1=yes, 0=no, 9=not indicated)

(a) post-conviction CPostC ___

(b) conditional discharge CCDisch ___

(c) pretrial diversion (adjourned in contemplation of dismissal) CPrtrial ___

(d) military disposition CMiltary ___

(e) other ___________________________ CSOther ___

55. Sample demographics for treatment comparison sample

(a) mean age (99 if missing) SMAge ______

(b) mean educational level (99 if missing) SMEduc ______

(c) % married (999 if missing) SPerM ______

(d) % African American (999 if missing) SPerAA ______

(e) % Hispanic (999 if missing) SPerHisp ______

(f) % employed (999 if missing) SPerEmp ______

(g) % with prior arrest (999 if missing) SPriorA ______

56. Was the abuse verified in some form (including conviction)?

(2=yes, 1=for some, 0=no, 9=not indicated) SVerify ___

57. Was the sample restricted in anyway (beyond exclusively using heterosexual intimate partners)? (1=yes, 0=no, 9=not indicated) SRstrct ___

58. If yes, indicate nature of restriction (Code 1=yes, 0=no, 8=n/a, 9=missing for each)

(a) age SRtrct01 ___

(b) language SRtrct02 ___

(c) geographical area SRtrct03 ___

(d) alcohol or drug dependence/abuse SRtrct04 ___

(e) mental illness SRtrct05 ___

(f) criminality SRtrct06 ___

(g) defendant had to agree SRtrct07 ___

(h) victim had to agree SRtrct08 ___

(i) judge had to agree SRtrct09 ___

(j) prosecutor had to agree SRtrct10 ___

(k) defense attorney had to agree SRtrct11 ___

(l) other __________________________________ SRtrct88 ___

59. Do any of the restrictions above result in a sample that is more likely to respond positively to treatment than the general population of domestic violence offenders? (i.e., is the sample “creamed” by eliminating unmotivated or otherwise difficult to treatment offenders?) (1=yes, 0=no, 8=not applicable, 9=cannot tell) SCream ___

60. What proportion of the population of domestic violence offenders in this jurisdiction is the sample? (999 if not indicated) SProp ______

61. Does the study examine (i.e., mention in the report) the relationship between treatment attendance (dose) and recidivism (1=yes, 0=no) DoseRel ___

62. If yes to #54, specify the nature of the observed relationship DoseEff ___

1. Negative (higher attendance, less recidivism), and statistically significant
2. Negative and statistically nonsignificant
3. Positive (higher attendance, higher recidivism), and statistically significant
4. Positive and statistically nonsignificant
5. Statistically nonsignificant, no direction reported
6. Statistically nonsignificant and correlation equals 0
7. Not applicable (i.e., answered no to 61)

**Outcome (Dependent Variable) Level Code Sheet**

Code the information below separately for each dependent variable (outcome) for which an effect size will be coded. Note that time of measurement is on the effect size level code sheet. As such, an outcome measured at multiple time points (rearrest at 6-months, 12-months, and 24-months) should only be coded once using this sheet.

**Identifying Information:**

63. Study (document) identifier StudyID ______

64. Dependent measure identifier DVID ______

65. Coder's initials DVCoder ______

66. Date coded DVDate ___ - __ - __

**Dependent Variable Information:**

67. Label _____________________________________________________________

68. Source of information DVSource ___

1. Official reports (police reports, etc.)

2. Victim report

1. Offender self-report
2. Victim report and offender self-report
3. Victim report and official report
4. Victim report, offender self-report and official report
5. Other _______________________________________

69. What is the variable measuring? DVCnstrt ___

1. domestic/partner abuse (same partner as initial offense resulting in entry into study)
2. domestic/partner abuse (same or other partner)
3. violent crime, excluding domestic/partner abuse
4. violent crime, includes domestic/partner abuse and other violent offenses (i.e., unspecified person offenses)
5. drug related offense
6. property offense

7. unspecified violation of probation (not specific to 1-5 above)

8. any type of offense (excluding technical violations)

9. any type of offense (including technical violations)

10. other behavioral _______________________________________

11. other nonbehavioral (attitudinal) ___________________________

70. If outcome includes technical violations, was failing to comply with treatment a violation of probation for the experimental conditions? (1=yes, 0=no, 8=not applicable, 9=cannot tell) DVViolate ___

71. For official report measures, indicate the nature of the indicator. (Code 1=yes, 0=no, 8=not applicable, 9=cannot tell for each of the categories below)

1. official complaint (complaint made to the police, probation officer

or judge; may or may not have resulted in an arrest) DVType1 ___

2. arrest DVType2 ___

3. conviction DVType3 ___

4. other ___________________ DVType4 ___

72. Level of measurement DVLOM ___

1. Dichotomous indicator

2. Frequency count

3. Composite scale (semi-continuous)

4. Other ________________________________

**Effect Size Level Coding Sheet**

Code this sheet separately for each eligible effect size.

**Identifying Information:**

73. Study (document) identifier StudyID ______

74. Treatment-Comparison identifier ESID ______

75. Outcome (dependent variable) identifier ESID(DVID) ______

76. Effect size identifier ESID (ESN) ______

77. Coder's initials ESCoder ______

78. Date coded ESDate ___ - __ - __

**Effect Size Related Information:**

79. Months from assignment to conditions to point of measurement. (Note: This represents the total time from the start of an individuals involvement in the study to the measurement point for this effect size. A 12-month post assignment to conditions indicator of recidivism would be coded as 12. A 12-month post the end of treatment would be coded 12+length of treatment. Record 99 if information is not available and 88 if not applicable)

(a) Same for all subjects TimeMon1 ____

(b) Average (if different across subjects) TimeMon2 ____

(c) Minimum (if different across subjects) TimeMon3 ____

(d) Maximum (if different across subjects) TimeMon4 ____

80. Time frame represented by measure in months. (Note: This represent the timeframe for the measure. For example, if arrest data were examined for a 12 month period, then record 12. Similarly, if a survey question asked about abuse during the past 30 days, record 1.)

(a) Same for all subjects ESTime1 ____

(b) Average (if different across subjects) ESTime2 ____

(c) Minimum (if different across subjects) ESTime3 ____

(d) Maximum (if different across subjects) ESTime4 ____

81. What Is the start of the time frame? StrtTime ___

1. Arrest

2. Conviction and/or Sentence

3. Assignment to conditions

4. Conviction and/or Sentence and/or Assignment to conditions

5. End of treatment for treatment group

6. End of treatment for individual subjects

8. Other ____________________

9. Cannot tell

82. Direction of effect. (Note: Specify the direction of the effect. Do not leave as missing or this effect size cannot be used.) ESDirect __

1. Effect favors experimental (treatment) condition

2. Effect favors control condition

3. Effect favors neither condition (no difference; effect size equals 0)

9. Cannot tell

**Effect Size Data---All Effect Sizes:**

83. Treatment group sample size ES_TxN _________

84. Control group sample size ES_CgN _________

**Effect Size Data---Continuous Type Measures:**

85. Treatment group mean ES_TxM _________

86. Control Group mean ES_CgM _________

87. Are the above means adjusted (e.g., ANCOVA adjusted)? (1=yes, 0=no)

ES_MAdj ___

88. Treatment group standard deviation ES_TxSD _________

89. Control group standard deviation ES_CgSD _________

90. Treatment group standard error ES_TxSE _________

91. Comparison group standard error ES_CgSE _________

92. *t*-value from an independent *t*-test or square root of *F*-value from a one-way analysis of variance with one *df* in the numerator (only two

groups) ES_t _________

**Effect Size Data---Dichotomous Measures:**

93. Treatment group; number of failures (recidivators) ES_TxNf_________

94. Comparison group; number failures (recidivators) ES_CgNf _________

95. Treatment group; proportion failures ES_TxPf _________

96. Comparison group; proportion failures ES_CgPf _________

97. Are the above proportions adjusted for pretest variables?

(1=yes; 0=no) ES_PAdj ___

98. Logged odds-ratio ES_LgOdd _________

99. Standard error of logged odds-ratio ES_SELgO_________

100. Logged odds-ratio adjusted? (e.g., from a logistic regression analysis with other independent variables)

(1=yes; 0=no) ES_OAdj _________

101. Chi-square value with *df* = 1 (2 by 2 contingency table)

ES_ChiSq _________

102. Correlation coefficient (phi) ES_RPhi _________

**Effect Size Data---Hand Calculated**:

103. Hand calculated *d*-type effect size ES_Hand1 _________

104. Hand calculated standard error of the *d*-type effect size

ES_Hand2 _________

# Appendix B: R Scripts for Data Analysis with Output

Below is the R-code for all analyses reported in this review. The data were coded directly into a relational database (FileMaker Pro) and exported to “dbf” files. The “dbf” files are read into R using the code below. There are 4 related data tables representing study-level variables (*study*), treatment or comparison-level variables (*tx*), outcome of dependent measure-level variables (*dv*), and effect size data (*es*). See codebook in Appedix B for additional details.

### Load needed libraries

library("foreign")
library("plyr")
library("dplyr")

##
## Attaching package: 'dplyr'

## The following objects are masked from 'package:plyr':
##
## arrange, count, desc, failwith, id, mutate, rename, summarise,
## summarize

## The following objects are masked from 'package:stats':
##
## filter, lag

## The following objects are masked from 'package:base':
##
## intersect, setdiff, setequal, union

library("magrittr")
library("metafor")

## Loading required package: Matrix

## Loading 'metafor' package (version 2.1-0). For an overview
## and introduction to the package please type: help(metafor).

library("knitr")
library("xtable")
library("kableExtra")

##
## Attaching package: 'kableExtra'

## The following object is masked from 'package:dplyr':
##
## group_rows

library("pander")
library("robumeta")

### Create some functions for doing computations

Below are functions used to compute the effect sizes. The FileMaker database also does these computations but these are reproduced (and validated) in R for transparency.

d2g <- function(d,n1,n2) {
 d * (1-(3/(4*(n1+n2)-9))) }
Cohensd <- function(m1,m2,s1,s2,n1,n2) {
 (m1-m2)/sqrt((s1^2*(n1-1) + s2^2*(n2-1)) / (n1+n2-2))
 }
Hedgesg <- function(m1,m2,s1,s2,n1,n2) {
 d <- Cohensd(m1,m2,s1,s2,n1,n2)
 g <- d2g(d,n1,n2)
 return(g)
 }
HedgesgV <- function(g,n1,n2) {
 (n1+n2)/(n1*n2) + (g^2/(2*(n1+n2)))
 }
OddsRatio <- function(a,b,c,d) {
 (a*d)/(b*c)
 }
LogOddsRatioFreq <- function(f1,n1,f2,n2) {
 a <- f1
 b <- n1 - a
 c <- f2
 d <- n2 - c
 return(log(OddsRatio(a,b,c,d)))
 }
LogOddsRatioProp <- function(p1,n1,p2,n2) {
 a <- p1*n1
 b <- n1 - a
 c <- p2*n2
 d <- n2 - c
 return(log(OddsRatio(a,b,c,d)))
 }
LogOddsRatioV <- function(a,b,c,d) {
 sqrt(1/a + 1/b + 1/c + 1/d)
 }
LogOddsRatioFreqV <- function(f1,n1,f2,n2) {
 a <- f1
 b <- n1 - a
 c <- f2
 d <- n2 - c
 return(LogOddsRatioV(a,b,c,d))
 }
LogOddsRatioPropV <- function(p1,n1,p2,n2) {
 a <- p1*n1
 b <- n1 - a
 c <- p2*n2
 d <- n2 - c
 return(LogOddsRatioV(a,b,c,d))
 }
g2lgor <- function(g) {g*1.65} # Cox conversion method
gv2lgorv <- function(v) {v*1.65^2} # Cox conversion method
lgor2g <- function(lgor) {lgor/1.65}
lgorv2gv <- function(lgorv) {lgorv/1.65^2}

### Read in the data

The code below reads in the four datafiles and does some preliminary cleanups.

setwd("~/new/dv/data/update2018/dbf")
study <- read.dbf("study.dbf", as.is = TRUE)
tx <- read.dbf("tx.dbf", as.is = TRUE)
dv <- read.dbf("dv.dbf", as.is = TRUE)
es <- read.dbf("es.dbf", as.is = TRUE)
setwd("~/new/dv/data/update2018/R")
## convert names to lowercase
names(study) <- tolower(names(study))
names(tx) <- tolower(names(tx))
names(dv) <- tolower(names(dv))
names(es) <- tolower(names(es))
## fix some fields to factors
for (i in 1:5) {
 es[ ,i] <- factor(es[ ,i])
}
## convert str to num for selected fields
for (i in 7:36) {
 es[ ,i] <- as.numeric(es[ ,i])
}
for (i in 6:13) {
 dv[ ,i] <- as.numeric(dv[ ,i])
}
## drop studies used in original review that are no longer eligible
## e.g., thouse with a drop-out control group
nrow(study)

## [1] 12

nrow(tx)

## [1] 16

nrow(dv)

## [1] 65

nrow(es)

## [1] 144

study <- subset(study, studyid!="33")
tx <- subset(tx, studyid!="33")
dv <- subset(dv, studyid!="33")
es <- subset(es, studyid!="33")
tx <- subset(tx, !(studyid=="27" & txid=="2"))
es <- subset(es, !(studyid=="27" & txid=="2"))

### Compute Effect Sizes and Associated Inverse Variance Weight

## determine available data for computations
es$dtype <- ifelse( complete.cases(es[ ,c("estxm","escgm","estxsd",
 "escgsd","estxn","escgn")]),
 1,
 ifelse( complete.cases(es[ ,c("eshand1","eshand2")]),
 2,
 ifelse( complete.cases(es[ ,c("eshand1")]),
 3,
 ifelse( complete.cases(es[ ,c("estxnf","escgnf","estxn","escgn")]),
 4,
 ifelse( complete.cases(es[ ,c("estxpf","escgpf","estxn","escgn")]),
 5,
 ifelse( complete.cases(es[ ,c("eslgodd","esselgo")]),
 6,7))))))
es$dtype <- factor(es$dtype, labels=c("Means/SDs","Hand with SE","Hand w/out SE",
 "2by2 freq","2by2 prop","Logistic Reg Coef"))
table(es$dtype)

##
## Means/SDs Hand with SE Hand w/out SE 2by2 freq
## 15 2 67 27
## 2by2 prop Logistic Reg Coef
## 27 2

## break es datafile apart
es1 <- es[es$dtype=="Means/SDs" , ]
es2 <- es[es$dtype=="Hand with SE", ]
es3 <- es[es$dtype=="Hand w/out SE", ]
es4 <- es[es$dtype=="2by2 freq", ]
es5 <- es[es$dtype=="2by2 prop", ]
es6 <- es[es$dtype=="Logistic Reg Coef", ]

## Compute ES based on means and SDs
es1$g <- with(es1, Hedgesg(estxm,escgm,estxsd,escgsd,estxn,escgn))
es1$gv <- with(es1, HedgesgV(g,estxn,escgn))
es1$lgor <- g2lgor(es1$g)
es1$lgorv <- gv2lgorv(es1$gv)

## Hand calculations provided Cohen's d
es2$g <- with(es2, d2g(eshand1,estxn,escgn))
es2$gv <- es2$eshand2
es2$lgor <- g2lgor(es2$g)
es2$lgorv <- gv2lgorv(es2$gv)

## Hand calculations provided Cohen's d
es3$g <- with(es3, d2g(eshand1,estxn,escgn))
es3$gv <- with(es3, HedgesgV(g,estxn,escgn))
es3$lgor <- g2lgor(es3$g)
es3$lgorv <- gv2lgorv(es3$gv)

## 2 by 2 Frequency
es4$lgor <- with(es4, LogOddsRatioFreq(estxnf,estxn,escgnf,escgn))
es4$lgorv <- with(es4, LogOddsRatioFreqV(estxnf,estxn,escgnf,escgn))
es4$g <- lgor2g(es4$lgor)
es4$gv <- lgorv2gv(es4$lgorv)

## 2 by 2 Proportions
es5$lgor <- with(es5, LogOddsRatioProp(estxpf,estxn,escgpf,escgn))
es5$lgorv <- with(es5, LogOddsRatioPropV(estxpf,estxn,escgpf,escgn))
es5$g <- lgor2g(es5$lgor)
es5$gv <- lgorv2gv(es5$lgorv)

## Logistic regression
es6$lgor <- es6$eslgodd
es6$lgorv <- es6$esselgo^2
es6$g <- lgor2g(es6$lgor)
es6$gv <- lgorv2gv(es6$lgorv)

## Put es dataframe back together
es <- rbind(es1,es2,es3,es4,es5,es6)
#es <- es[ ,-c(19,20,22:28,30,31,33:36, 38:44)]

## Fix direction of Effect Size
es$g <- ifelse(es$esdirect==1 & es$g<0, es$g*-1, es$g)
es$g <- ifelse(es$esdirect==2 & es$g>0, es$g*-1, es$g)
es$g <- ifelse(es$esdirect==3, 0, es$g)
es$lgor <- ifelse(es$esdirect==1 & es$lgor>0, es$lgor*-1, es$lgor)
es$lgor <- ifelse(es$esdirect==2 & es$lgor<0, es$lgor*-1, es$lgor)
es$lgor <- ifelse(es$esdirect==3, 0, es$lgor)
aggregate(g ~ esdirect, data=es, summary)

## esdirect g.Min. g.1st Qu. g.Median g.Mean
## 1 1 0.0009973936 0.0657616293 0.1445224461 0.2191678641
## 2 2 -1.0367145531 -0.1670857338 -0.1231686313 -0.1438134862
## 3 3 0.0000000000 0.0000000000 0.0000000000 0.0000000000
## g.3rd Qu. g.Max.
## 1 0.2643917327 1.6803568014
## 2 -0.0380063479 -0.0009974811
## 3 0.0000000000 0.0000000000

aggregate(lgor~esdirect, data=es, summary)

## esdirect lgor.Min. lgor.1st Qu. lgor.Median lgor.Mean lgor.3rd Qu.
## 1 1 -2.772588722 -0.436246359 -0.238462036 -0.361626976 -0.108506688
## 2 2 0.001645844 0.062710474 0.203228242 0.237292252 0.275691461
## 3 3 0.000000000 0.000000000 0.000000000 0.000000000 0.000000000
## lgor.Max.
## 1 -0.001645699
## 2 1.710579013
## 3 0.000000000

### Merge Data into a Single Dataframe

Create a single dataframe that has one row per effect size but all of the data from the study, tx, and dv dataframes.

## merge data
fulldata <- join(es,dv,by=c("studyid","dvid"), match="all")
fulldata <- join(fulldata,tx,by=c("studyid","txid"), match="all")
fulldata <- join(fulldata,study,by="studyid", match="all")
nrow(fulldata)

## [1] 140

### Create Subset of Effect Size for Official Measures of Domestic Violence

## official reports violence
official <- subset(fulldata,
 (dvsource==1 | dvsource==5 | dvsource==6) & dvcnstrt<3)
nrow(official)

## [1] 21

## 105 only has general violence but not DV violence; add it to above file
official <- rbind(official, fulldata[ fulldata$studyid=="105" & fulldata$dvcnstrt==4, ])
nrow(official)

## [1] 22

table(official$studyid,official$txid)

##
## 1 2 3
## 1 1 1 1
## 101 1 0 0
## 105 1 0 0
## 12 1 0 0
## 157 2 0 0
## 2 2 2 0
## 27 2 0 0
## 30 2 0 0
## 33 0 0 0
## 4 1 0 0
## 45 4 0 0
## 5 1 0 0

## note: 5 studies have multiple official measures
## get to one ES per study
## preferences
### arrest > conviction
### baseline adjusted > not
### longer time frame
official <- subset(official, !(studyid=="2" & timemon1==6))
official <- subset(official, !(studyid=="27" & dvid=="2"))
official <- subset(official, !(studyid=="27" & dvid=="2"))
official <- subset(official, !(studyid=="30" & dvid=="6"))
official <- subset(official, !(studyid=="33" & espadj!=1)) ##
official <- subset(official, !(studyid=="45" & timemon1==6))
official <- subset(official, !(studyid=="45" & esoadj!=1))
official <- subset(official, !(studyid=="157" & estime1>12))
nrow(official) ## good, one effect size per study/tx comparison

## [1] 14

table(official$studyid,official$txid)

##
## 1 2 3
## 1 1 1 1
## 101 1 0 0
## 105 1 0 0
## 12 1 0 0
## 157 1 0 0
## 2 1 1 0
## 27 1 0 0
## 30 1 0 0
## 33 0 0 0
## 4 1 0 0
## 45 1 0 0
## 5 1 0 0

### Create Table 1

Below is the code to create Table 1.

studytx <- join(tx, study, by=c("studyid"), match="all")
studytx$txweeks <- as.character(studytx$txweeks)
studytx$txweeks[studytx$txweeks=="99"] <- "Missing"
studytx$txnum <- as.character(studytx$txnum)
studytx$txnum[studytx$txnum=="99"] <- "Missing"
studytx$txhours2 <- as.character(studytx$txhours2)
studytx$txhours2[studytx$txhours2=="99"] <- "Missing"
studytx$length <- with(studytx, paste(txweeks,txnum,txhours2,sep="/"))
studytx$txcompl <- paste0(as.character(studytx$txcompl),"%")
studytx$txcompl[studytx$txcompl=="999%"] <- "Missing"
#studytx$random[studytx$txrandom<4] <- "Random"
#studytx$random[studytx$txrandom==4] <- "Quasi-Experimental"
studytx$random[studytx$txrandom==1] <- "Random"
studytx$random[studytx$txrandom>1] <- "Quasi-Experimental"
studytx <- arrange(studytx, desc(random), forestplot)
table1 <- with(studytx, cbind(forestplot,txlabel,length,txcompl,cglabel,samplelbl))
table1 <- as.data.frame(table1)
names(table1) <- c("Author/Year",
 "Program Type",
 "Program Weeks/Sessions/Hours",
 "% Completing Program",
 "Control Type",
 "Sample")
#table1
write.table(table1, file = "table1.txt", sep = "\t", quote = FALSE, row.names = F)

### Create Table 2

Below is the code to create Table 2.

studytx <- join(tx, study, by=c("studyid"), match="all")
studytx$Ns <- paste(as.character(studytx$stxn),as.character(studytx$scgn),sep="/")
studytx <- join(studytx,official[ ,c("studyid","txid","esattrit")],
 by=c("studyid","txid"), match="all")
studytx$esattrit[ studytx$esattrit>1] <- 1
studytx$esattrit <- paste0(as.character(studytx$esattrit*100),"%")
studytx$itt <- case_when(
 studytx$txanalyz == 1 ~ "Intent-to-treat",
 studytx$txanalyz == 4 ~ "Treated",
 studytx$txanalyz == 8 ~ "Treated"
)
studytx$random[studytx$txrandom==1] <- "Random"
studytx$random[studytx$txrandom>1] <- "Quasi-Experimental"
studytx <- arrange(studytx, desc(random), forestplot)
table2 <- with(studytx, cbind(forestplot,designlbl,Ns,itt,selectbias,esattrit,missingdvs))
table2 <- as.data.frame(table2)
names(table2) <- c("Author/Year",
 "Research Design",
 "Treatment/Control Sample Size",
 "How analyzed",
 "Risk of Selection Bias", "Attrition (Main Outcome)",
 "Selective Reporting of Outcomes"
 )
#table2
write.table(table2, file = "table2.txt", sep = "\t", quote = FALSE, row.names = F)

### Run Analysis of Official Measures of Recidivism

Run analyses of official measures of recividism for the random assignment condition and the quasi-experimental condition. Create Table 3 summarizig the results.

## run meta-analyses
official_random <- subset(official, txrandom==1)
official_quasi <- subset(official, txrandom>1)

## analysis of RCTs (official measures)
randmodel_official <- rma(lgor, lgorv, method="DL", data=official_random)
summary(randmodel_official)

##
## Random-Effects Model (k = 7; tau^2 estimator: DL)
##
## logLik deviance AIC BIC AICc
## -5.4943 3.8323 14.9886 14.8804 17.9886
##
## tau^2 (estimated amount of total heterogeneity): 0 (SE = 0.2475)
## tau (square root of estimated tau^2 value): 0
## I^2 (total heterogeneity / total variability): 0.00%
## H^2 (total variability / sampling variability): 1.00
##
## Test for Heterogeneity:
## Q(df = 6) = 3.8323, p-val = 0.6994
##
## Model Results:
##
## estimate se zval pval ci.lb ci.ub
## -0.2364 0.2456 -0.9628 0.3357 -0.7177 0.2449
##
## ---
## Signif. codes: 0 '***' 0.001 '**' 0.01 '*' 0.05 '.' 0.1 ' ' 1

forest(randmodel_official, xlab="Odds Ratio",
 atransf=exp, annotate=FALSE, slab=official_random$forestplot,col="#eee8d5")
text(-6, 8.5, "Author(s) and Year", pos=4)
text(0, 8.5, "Favors Treatment", pos=2)
text(0, 8.5, "Favors Control", pos=4)


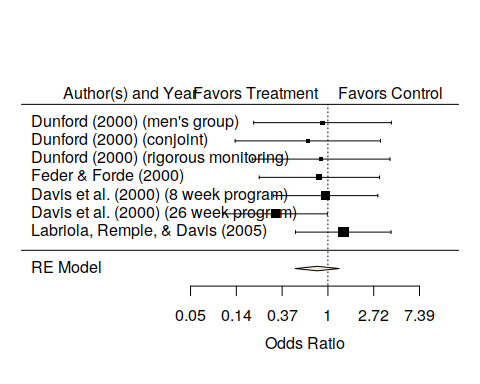


png('random.png', width=800,height=450)
forest(randmodel_official, xlab="Odds Ratio",
 atransf=exp, annotate=FALSE, slab=official_random$forestplot,col="#eee8d5")
text(-6, 8.5, "Author(s) and Year", pos=4)
text(0, 8.5, "Favors Treatment", pos=2)
text(0, 8.5, "Favors Control", pos=4)
dev.off()

## png
## 2

## analysis of Quasi-Experiments (official measures)
official_quasi$forestplot <- recode(official_quasi$forestplot,
 "Gordon & Moriarity (2003) (mandated vs. not)" = "Gordon & Moriarity (2003)")
quasimodel_official <- rma(lgor, lgorv, method="DL", data=official_quasi)
summary(quasimodel_official)

##
## Random-Effects Model (k = 7; tau^2 estimator: DL)
##
## logLik deviance AIC BIC AICc
## -10.5690 14.7051 25.1379 25.0297 28.1379
##
## tau^2 (estimated amount of total heterogeneity): 0.7920 (SE = 0.6985)
## tau (square root of estimated tau^2 value): 0.8899
## I^2 (total heterogeneity / total variability): 68.30%
## H^2 (total variability / sampling variability): 3.15
##
## Test for Heterogeneity:
## Q(df = 6) = 18.9291, p-val = 0.0043
##
## Model Results:
##
## estimate se zval pval ci.lb ci.ub
## -0.6113 0.4151 -1.4726 0.1409 -1.4250 0.2023
##
## ---
## Signif. codes: 0 '***' 0.001 '**' 0.01 '*' 0.05 '.' 0.1 ' ' 1

forest(quasimodel_official, xlab="Odds Ratio",
 atransf=exp, annotate=FALSE, slab=official_quasi$forestplot,col="#eee8d5")
text(-12, 8.5, "Author(s) and Year", pos=4)
text(0, 8.5, "Favors Treatment", pos=2)
text(0, 8.5, "Favors Control", pos=4)


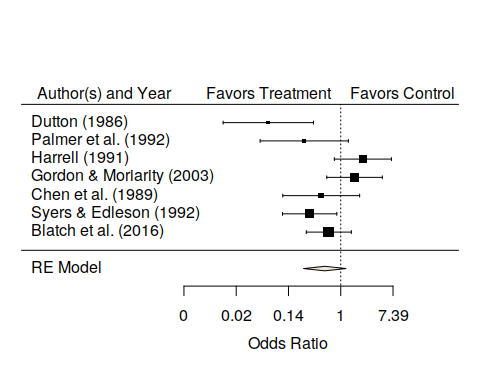


png('quasi.png', width=800,height=450)
forest(quasimodel_official, xlab="Odds Ratio",
 atransf=exp, annotate=FALSE, slab=official_quasi$forestplot,col="#eee8d5")
text(-12, 8.5, "Author(s) and Year", pos=4)
text(0, 8.5, "Favors Treatment", pos=2)
text(0, 8.5, "Favors Control", pos=4)
dev.off()

## png
## 2

## analysis of both RCTs and Quasi-Experiments (official measures)
allmodel_official <- rma(lgor, lgorv, method="DL", data=official)
summary(allmodel_official)

##
## Random-Effects Model (k = 14; tau^2 estimator: DL)
##
## logLik deviance AIC BIC AICc
## -17.5289 21.4688 39.0579 40.3360 40.1488
##
## tau^2 (estimated amount of total heterogeneity): 0.3053 (SE = 0.2771)
## tau (square root of estimated tau^2 value): 0.5525
## I^2 (total heterogeneity / total variability): 43.88%
## H^2 (total variability / sampling variability): 1.78
##
## Test for Heterogeneity:
## Q(df = 13) = 23.1649, p-val = 0.0397
##
## Model Results:
##
## estimate se zval pval ci.lb ci.ub
## -0.3987 0.2268 -1.7579 0.0788 -0.8432 0.0458 .
##
## ---
## Signif. codes: 0 '***' 0.001 '**' 0.01 '*' 0.05 '.' 0.1 ' ' 1

allmodel_official_design_moderator <-
 rma(lgor ~ txrandom==1, lgorv, method="DL", data=official)
summary(allmodel_official_design_moderator)

##
## Mixed-Effects Model (k = 14; tau^2 estimator: DL)
##
## logLik deviance AIC BIC AICc
## -17.3710 21.1530 40.7421 42.6592 43.1421
##
## tau^2 (estimated amount of residual heterogeneity): 0.3549 (SE = 0.3116)
## tau (square root of estimated tau^2 value): 0.5958
## I^2 (residual heterogeneity / unaccounted variability): 47.28%
## H^2 (unaccounted variability / sampling variability): 1.90
## R^2 (amount of heterogeneity accounted for): 0.00%
##
## Test for Residual Heterogeneity:
## QE(df = 12) = 22.7614, p-val = 0.0298
##
## Test of Moderators (coefficient 2):
## QM(df = 1) = 0.4271, p-val = 0.5134
##
## Model Results:
##
## estimate se zval pval ci.lb ci.ub
## intrcpt -0.5528 0.3275 -1.6880 0.0914 -1.1946 0.0891 .
## txrandom == 1TRUE 0.3071 0.4699 0.6535 0.5134 -0.6139 1.2282
##
## ---
## Signif. codes: 0 '***' 0.001 '**' 0.01 '*' 0.05 '.' 0.1 ' ' 1

## victim report of violaence
victim_all <- subset(fulldata, (dvsource==2 | dvsource==4) & dvcnstrt<3)
nrow(victim_all)

## [1] 61

## determine longest follow-up within DV construct
victim_all$timemon1[(victim_all$timemon1==88 | victim_all$timemon1==99)] <- NA
victim_all$timemon2[(victim_all$timemon2==88 | victim_all$timemon2==99)] <- NA
victim_all$timemon3[(victim_all$timemon3==88 | victim_all$timemon3==99)] <- NA
victim_all$timemon4[(victim_all$timemon4==88 | victim_all$timemon4==99)] <- NA
longest <- victim_all %>%
 group_by(studyid, txid, dvid) %>%
 summarize(timemon1max = max(timemon1),
 timemon2max = max(timemon2),
 timemon3max = max(timemon3),
 timemon4max = max(timemon4))
victim_all <- merge(victim_all, longest, by=c("studyid","txid","dvid"))
victim_all <- subset(victim_all, (!is.na(timemon1) & timemon1==timemon1max) |
 (!is.na(timemon2) & timemon2==timemon2max) |
 (!is.na(timemon3) & timemon2==timemon3max) |
 (!is.na(timemon4) & timemon2==timemon4max) |
 (is.na(timemon1) & is.na(timemon2) &
 is.na(timemon3) & is.na(timemon4)))
nrow(victim_all)

## [1] 54

## average multiple effect sizes
lgor <- aggregate(lgor ~ studyid+txid+forestplot+txrandom, data=victim_all, mean)
lgorv <- aggregate(lgorv ~ studyid+txid+forestplot+txrandom, data=victim_all, mean)
victim <- cbind(lgor,lgorv=lgorv$lgorv)
names(victim)

## [1] "studyid" "txid" "forestplot" "txrandom" "lgor"
## [6] "lgorv"

victim_random <- victim[ victim$txrandom==1, ]
victim_quasi <- victim[ victim$txrandom>1, ]
## perform meta-analyses
## randomized designs
randmodel_victim <- rma(lgor, lgorv, method="DL", data=victim_random)
summary(randmodel_victim)

##
## Random-Effects Model (k = 7; tau^2 estimator: DL)
##
## logLik deviance AIC BIC AICc
## -1.2961 1.0306 6.5922 6.4840 9.5922
##
## tau^2 (estimated amount of total heterogeneity): 0 (SE = 0.0960)
## tau (square root of estimated tau^2 value): 0
## I^2 (total heterogeneity / total variability): 0.00%
## H^2 (total variability / sampling variability): 1.00
##
## Test for Heterogeneity:
## Q(df = 6) = 1.0306, p-val = 0.9844
##
## Model Results:
##
## estimate se zval pval ci.lb ci.ub
## -0.0113 0.1473 -0.0765 0.9390 -0.2999 0.2774
##
## ---
## Signif. codes: 0 '***' 0.001 '**' 0.01 '*' 0.05 '.' 0.1 ' ' 1

## quasi experimental
quasimodel_victim <- rma(lgor, lgorv, method="FE", data=victim_quasi)
summary(quasimodel_victim)

##
## Fixed-Effects Model (k = 1)
##
## logLik deviance AIC BIC AICc
## -0.4683 0.0000 2.9366 0.9366 6.9366
##
## Test for Heterogeneity:
## Q(df = 0) = 0.0000, p-val = 1.0000
##
## Model Results:
##
## estimate se zval pval ci.lb ci.ub
## 0.5655 0.6372 0.8875 0.3748 -0.6834 1.8145
##
## ---
## Signif. codes: 0 '***' 0.001 '**' 0.01 '*' 0.05 '.' 0.1 ' ' 1

## randomized designs
all_victim <- rma(lgor, lgorv, method="DL", data=victim)
summary(all_victim)

##
## Random-Effects Model (k = 8; tau^2 estimator: DL)
##
## logLik deviance AIC BIC AICc
## -2.1533 1.8083 8.3066 8.4655 10.7066
##
## tau^2 (estimated amount of total heterogeneity): 0 (SE = 0.0961)
## tau (square root of estimated tau^2 value): 0
## I^2 (total heterogeneity / total variability): 0.00%
## H^2 (total variability / sampling variability): 1.00
##
## Test for Heterogeneity:
## Q(df = 7) = 1.8083, p-val = 0.9697
##
## Model Results:
##
## estimate se zval pval ci.lb ci.ub
## 0.0180 0.1435 0.1253 0.9003 -0.2633 0.2992
##
## ---
## Signif. codes: 0 '***' 0.001 '**' 0.01 '*' 0.05 '.' 0.1 ' ' 1

all_victim_design <- rma(lgor ~ txrandom==1, lgorv, method="DL", data=victim)
summary(all_victim_design)

##
## Mixed-Effects Model (k = 8; tau^2 estimator: DL)
##
## logLik deviance AIC BIC AICc
## -1.7644 1.0306 9.5288 9.7671 15.5288
##
## tau^2 (estimated amount of residual heterogeneity): 0 (SE = 0.0960)
## tau (square root of estimated tau^2 value): 0
## I^2 (residual heterogeneity / unaccounted variability): 0.00%
## H^2 (unaccounted variability / sampling variability): 1.00
## R^2 (amount of heterogeneity accounted for): 0.00%
##
## Test for Residual Heterogeneity:
## QE(df = 6) = 1.0306, p-val = 0.9844
##
## Test of Moderators (coefficient 2):
## QM(df = 1) = 0.7778, p-val = 0.3778
##
## Model Results:
##
## estimate se zval pval ci.lb ci.ub
## intrcpt 0.5655 0.6372 0.8875 0.3748 -0.6834 1.8145
## txrandom == 1TRUE -0.5768 0.6540 -0.8819 0.3778 -1.8587 0.7051
##
## ---
## Signif. codes: 0 '***' 0.001 '**' 0.01 '*' 0.05 '.' 0.1 ' ' 1

## forest plots
forest(randmodel_victim, xlab="Odds Ratio",
 atransf=exp, annotate=FALSE, slab=victim_random$forestplot,col="#eee8d5")
text(-5, 8.5, "Author(s) and Year", pos=4)
text(0, 8.5, "Favors Treatment", pos=2)
text(0, 8.5, "Favors Control", pos=4)


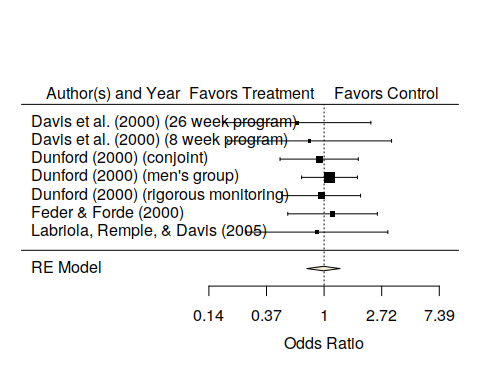


png('rand_victim.png', width=800,height=450)
forest(randmodel_victim, xlab="Odds Ratio",
 atransf=exp, annotate=FALSE, slab=victim_random$forestplot,col="#eee8d5")
text(-5, 8.5, "Author(s) and Year", pos=4)
text(0, 8.5, "Favors Treatment", pos=2)
text(0, 8.5, "Favors Control", pos=4)
dev.off()

## png
## 2

forest(quasimodel_victim, xlab="Odds Ratio",
 atransf=exp, annotate=FALSE, slab=victim_quasi$forestplot,col="#eee8d5")
text(-3.5, 2.5, "Author(s) and Year", pos=4)
text(0, 2.5, "Favors Treatment", pos=2)
text(0, 2.5, "Favors Control", pos=4)


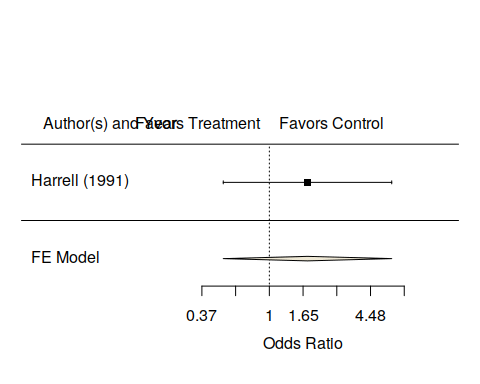


png('quasi_victim.png', width=800,height=450)
forest(quasimodel_victim, xlab="Odds Ratio",
 atransf=exp, annotate=FALSE, slab=victim_quasi$forestplot,col="#eee8d5")
text(-3.5, 2.5, "Author(s) and Year", pos=4)
text(0, 2.5, "Favors Treatment", pos=2)
text(0, 2.5, "Favors Control", pos=4)
dev.off()

## png
## 2

##
## create table 3
##
## table columns
## 1 = Analysis
## 2 = Odds ratio
## 3 = Lower 95% Odds ratio
## 4 = Upper 95% Odds ratio
## 5 = Q (from FE model)
## 6 = p(Q) (from FE model)
## 7 = tau^2
## 8 = k
maResultsRE <- function(model) {
 OR <- exp(model$b)
 LOR <- exp(model$ci.lb)
 UOR <- exp(model$ci.ub)
 tau2 <- model$tau2
 k <- model$k
 rowdata <- cbind.data.frame(OR,LOR,UOR,tau2,k)
 return(rowdata)
}
maResultsFE <- function(model) {
 Q <- model$QE
 Qp <- model$QEp
 rowdata <- cbind.data.frame(Q,Qp)
 return(rowdata)
}
row1 <- cbind("Analysis" = "Official -- RCT",
 maResultsRE(randmodel_official),maResultsFE(randmodel_official))
row2 <- cbind("Analysis" = "Official -- QE" ,
 maResultsRE(quasimodel_official),maResultsFE(quasimodel_official))
row3 <- cbind("Analysis" = "Official -- RCT & QE" ,
 maResultsRE(allmodel_official),maResultsFE(allmodel_official))
row4 <- cbind("Analysis" = "Victim -- RCT" ,
 maResultsRE(randmodel_victim),maResultsFE(randmodel_victim))
row5 <- cbind("Analysis" = "Victim -- QE" ,
 maResultsRE(quasimodel_victim),maResultsFE(quasimodel_victim))
row6 <- cbind("Analysis" = "Victim -- RCT & QE" ,
 maResultsRE(all_victim),maResultsFE(all_victim))
table3 <- rbind(row1,row2,row3,row4,row5,row6)
names(table3) <- c("Analysis", "Odds ratio", "Lower 95% CI", "Upper 95% CI", "Tau^2", "k","Q","p of Q")
table3$i2 <- ((table3$Q - (table3$k-1))/table3$Q) * 100
table3$i2[table3$i2<0] <- NaN
table3 <- table3[ , c(1:4,7:8,9,5:6)]
table3[, 2] <- round(table3[ ,2], digits = 2)
table3[, 3] <- round(table3[ ,3], digits = 2)
table3[, 4] <- round(table3[ ,4], digits = 2)
table3[, 5] <- round(table3[ ,5], digits = 3)
table3[, 6] <- round(table3[ ,6], digits = 3)
table3[, 7] <- round(table3[ ,7], digits = 0)
table3[, 8] <- round(table3[ ,8], digits = 3)
write.table(table3, file = "table3.txt", sep = "\t", quote = FALSE, row.names = F)

### Re-run analyses of random assignment studies

Re-run random assignment analyses dropping Dunford’s conjoint and rigorous monitoring (keeping the men’s only group) and combining the two Davis groups (8 and 26 weeks)

# break the data apart (official data)
dunford <- subset(official_random, author=="Dunford (2000)")
dunford <- subset(dunford, txid==1)
davis <- subset(official_random, substr(author,1,5)=="Davis")
official_random_subset <- subset(official_random, author!="Dunford (2000)" &
 substr(author,1,5)!="Davis")
## collapse Davis, adding together the 8-week and 26-week outcomes
davis <- davis[ ,c("studyid","dvid","estxn","escgn","author","estxpf","escgpf")]
davis$estxf <- davis$estxn * davis$estxpf
davis <- davis %>%
 group_by(studyid, dvid, escgn, author, escgpf) %>%
 summarize(estxn = sum(estxn), estxf = sum(estxf))
davis <- as.data.frame(davis)
davis$estxpdf <- davis$estxf / davis$estxn
davis$lgor <- with(davis, LogOddsRatioProp(estxpdf, estxn, escgpf, escgn))
davis$lgorv <- with(davis, LogOddsRatioPropV(estxpdf, estxn, escgpf, escgn))
davis$forestplot <- davis$author
## put the data back together
official_random_subset <- rbind.fill(official_random_subset, dunford, davis)
official_random_subset <- official_random_subset[ order(official_random_subset$author), ]
## run analysis and create forest plot
randmodel_official_subset <- rma(lgor, lgorv, method="DL", data=official_random_subset)
summary(randmodel_official_subset)

##
## Random-Effects Model (k = 4; tau^2 estimator: DL)
##
## logLik deviance AIC BIC AICc
## -2.7020 1.9764 9.4041 8.1767 21.4041
##
## tau^2 (estimated amount of total heterogeneity): 0 (SE = 0.3001)
## tau (square root of estimated tau^2 value): 0
## I^2 (total heterogeneity / total variability): 0.00%
## H^2 (total variability / sampling variability): 1.00
##
## Test for Heterogeneity:
## Q(df = 3) = 1.9764, p-val = 0.5773
##
## Model Results:
##
## estimate se zval pval ci.lb ci.ub
## -0.1867 0.2982 -0.6261 0.5312 -0.7712 0.3978
##
## ---
## Signif. codes: 0 '***' 0.001 '**' 0.01 '*' 0.05 '.' 0.1 ' ' 1

#par(bg="#002b36",fg="#eee8d5")
forest(randmodel_official_subset, xlab="Odds Ratio",
 atransf=exp, annotate=FALSE, slab=official_random_subset$forestplot,col="#eee8d5")
text(-5, 5.5, "Author(s) and Year", pos=4)
text(0, 5.5, "Favors Treatment", pos=2)
text(0, 5.5, "Favors Control", pos=4)


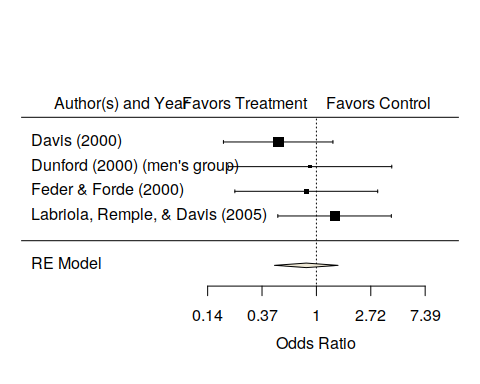


png('rand_official_subset.png', width=800,height=450)
#par(bg="#002b36",fg="#eee8d5")
forest(randmodel_official_subset, xlab="Odds Ratio",
 atransf=exp, annotate=FALSE, slab=official_random_subset$forestplot,col="#eee8d5")
text(-5, 5.5, "Author(s) and Year", pos=4)
text(0, 5.5, "Favors Treatment", pos=2)
text(0, 5.5, "Favors Control", pos=4)
dev.off()

## png
## 2

# victim data without Davis (studyid 2) and dropping
# Dunford conjoint and rigorous monitoring (txids 2 and 3; all others are txid 1)
victim_random_subset <- subset(victim_random, studyid!=2 & txid==1)
# get Davis data
davis_victim <- subset(victim_all,
 substr(author,1,5)=="Davis" & (dvsource==2 | dvsource==4) & dvcnstrt<3)
davis_victim <- davis_victim[ ,c("studyid","dvid","estxn","escgn","author","estxpf","escgpf")]
davis_victim$estxf <- davis_victim$estxn * davis_victim$estxpf
davis_victim <- davis_victim %>%
 group_by(studyid, dvid, escgn, author, escgpf) %>%
 summarize(estxn = sum(estxn), estxf = sum(estxf))
davis_victim <- as.data.frame(davis_victim)
davis_victim$estxpdf <- davis_victim$estxf / davis_victim$estxn
davis_victim$lgor <- with(davis_victim, LogOddsRatioProp(estxpdf, estxn, escgpf, escgn))
davis_victim$lgorv <- with(davis_victim, LogOddsRatioPropV(estxpdf, estxn, escgpf, escgn))
davis_victim$forestplot <- davis_victim$author
## put the data back together
victim_random_subset <- rbind.fill(victim_random_subset, davis_victim)
victim_random_subset <- victim_random_subset[ order(victim_random_subset$author), ]
## run analysis and create forest plot
randmodel_victim_subset <- rma(lgor, lgorv, method="DL", data=victim_random_subset)
summary(randmodel_victim_subset)

##
## Random-Effects Model (k = 4; tau^2 estimator: DL)
##
## logLik deviance AIC BIC AICc
## -0.7410 0.6913 5.4820 4.2546 17.4820
##
## tau^2 (estimated amount of total heterogeneity): 0 (SE = 0.1492)
## tau (square root of estimated tau^2 value): 0
## I^2 (total heterogeneity / total variability): 0.00%
## H^2 (total variability / sampling variability): 1.00
##
## Test for Heterogeneity:
## Q(df = 3) = 0.6913, p-val = 0.8752
##
## Model Results:
##
## estimate se zval pval ci.lb ci.ub
## 0.0402 0.1892 0.2126 0.8316 -0.3306 0.4111
##
## ---
## Signif. codes: 0 '***' 0.001 '**' 0.01 '*' 0.05 '.' 0.1 ' ' 1

forest(randmodel_victim_subset, xlab="Odds Ratio",
 atransf=exp, annotate=FALSE, slab=victim_random_subset$forestplot,col="#eee8d5")
text(-4.5, 5.5, "Author(s) and Year", pos=4)
text(0, 5.5, "Favors Treatment", pos=2)
text(0, 5.5, "Favors Control", pos=4)


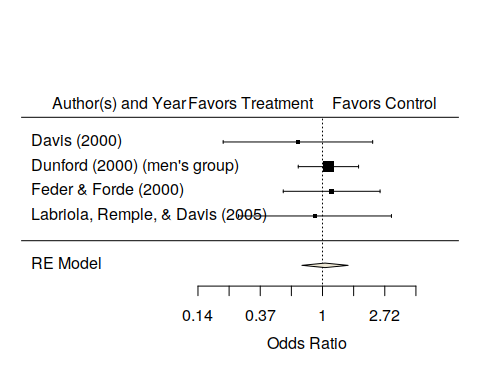


png('random_victim_subset.png', width=800,height=450)
#par(bg="#002b36",fg="#eee8d5")
forest(randmodel_victim_subset, xlab="Odds Ratio",
 atransf=exp, annotate=FALSE, slab=victim_random_subset$forestplot,col="#eee8d5")
text(-4.5, 5.5, "Author(s) and Year", pos=4)
text(0, 5.5, "Favors Treatment", pos=2)
text(0, 5.5, "Favors Control", pos=4)
dev.off()

## png
## 2

### Publication Selection Bias

Below we examine the forest plot and trim-and-fill analysis for official measures by design type (RCT and QE). We also run these analyses for the combined RCT+QE datasets. Finally we run a meta-regression model comparing published to unpublished studies using the combined RCT+QE datasets.

m1 <- trimfill(randmodel_official, side="right")
m1

##
## Estimated number of missing studies on the right side: 0 (SE = 1.6454)
##
## Random-Effects Model (k = 7; tau^2 estimator: DL)
##
## tau^2 (estimated amount of total heterogeneity): 0 (SE = 0.2475)
## tau (square root of estimated tau^2 value): 0
## I^2 (total heterogeneity / total variability): 0.00%
## H^2 (total variability / sampling variability): 1.00
##
## Test for Heterogeneity:
## Q(df = 6) = 3.8323, p-val = 0.6994
##
## Model Results:
##
## estimate se zval pval ci.lb ci.ub
## -0.2364 0.2456 -0.9628 0.3357 -0.7177 0.2449
##
## ---
## Signif. codes: 0 '***' 0.001 '**' 0.01 '*' 0.05 '.' 0.1 ' ' 1

funnel(m1)


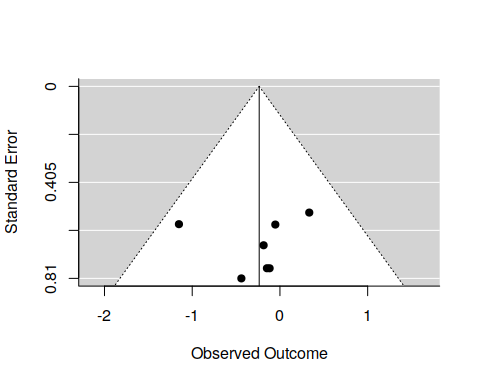


m2 <- trimfill(quasimodel_official, side="right")
m2

##
## Estimated number of missing studies on the right side: 0 (SE = 1.8715)
##
## Random-Effects Model (k = 7; tau^2 estimator: DL)
##
## tau^2 (estimated amount of total heterogeneity): 0.7920 (SE = 0.6985)
## tau (square root of estimated tau^2 value): 0.8899
## I^2 (total heterogeneity / total variability): 68.30%
## H^2 (total variability / sampling variability): 3.15
##
## Test for Heterogeneity:
## Q(df = 6) = 18.9291, p-val = 0.0043
##
## Model Results:
##
## estimate se zval pval ci.lb ci.ub
## -0.6113 0.4151 -1.4726 0.1409 -1.4250 0.2023
##
## ---
## Signif. codes: 0 '***' 0.001 '**' 0.01 '*' 0.05 '.' 0.1 ' ' 1

funnel(m2)


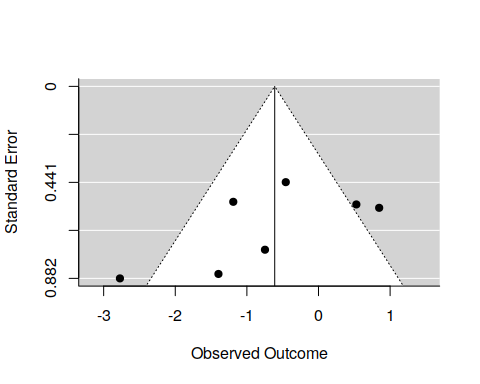


maofficial <- rma(lgor, lgorv, method="DL", data=official)
m3 <- trimfill(maofficial, side="right")
png('funnel1.png', width=800,height=450)
funnel(m3)
dev.off()

## png
## 2

funnel(m3)


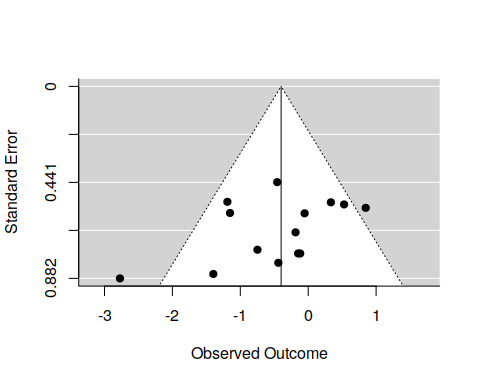


table(official$pubtype)

##
## 3 4 5 7
## 9 3 1 1

official$published[official$pubtype==3] <- 1
official$published[official$pubtype!=3] <- 0
ma_pubtype <- rma(lgor ~ published, lgorv, method="DL", data=official)
print(ma_pubtype)

##
## Mixed-Effects Model (k = 14; tau^2 estimator: DL)
##
## tau^2 (estimated amount of residual heterogeneity): 0.2692 (SE = 0.2752)
## tau (square root of estimated tau^2 value): 0.5188
## I^2 (residual heterogeneity / unaccounted variability): 40.42%
## H^2 (unaccounted variability / sampling variability): 1.68
## R^2 (amount of heterogeneity accounted for): 11.83%
##
## Test for Residual Heterogeneity:
## QE(df = 12) = 20.1417, p-val = 0.0645
##
## Test of Moderators (coefficient 2):
## QM(df = 1) = 1.9071, p-val = 0.1673
##
## Model Results:
##
## estimate se zval pval ci.lb ci.ub
## intrcpt -0.0216 0.3489 -0.0618 0.9507 -0.7053 0.6622
## published -0.6220 0.4504 -1.3810 0.1673 -1.5049 0.2608
##
## ---
## Signif. codes: 0 '***' 0.001 '**' 0.01 '*' 0.05 '.' 0.1 ' ' 1

published <- subset(official, published==1)
unpublished <- subset(official, published==0)
ma_published <- rma(lgor, lgorv, method="DL", data=published)
ma_unpublished <- rma(lgor, lgorv, method="DL", data=unpublished)
print(ma_published)

##
## Random-Effects Model (k = 9; tau^2 estimator: DL)
##
## tau^2 (estimated amount of total heterogeneity): 0.2949 (SE = 0.3716)
## tau (square root of estimated tau^2 value): 0.5431
## I^2 (total heterogeneity / total variability): 40.43%
## H^2 (total variability / sampling variability): 1.68
##
## Test for Heterogeneity:
## Q(df = 8) = 13.4297, p-val = 0.0979
##
## Model Results:
##
## estimate se zval pval ci.lb ci.ub
## -0.6468 0.2903 -2.2277 0.0259 -1.2158 -0.0777 *
##
## ---
## Signif. codes: 0 '***' 0.001 '**' 0.01 '*' 0.05 '.' 0.1 ' ' 1

print(ma_unpublished)

##
## Random-Effects Model (k = 5; tau^2 estimator: DL)
##
## tau^2 (estimated amount of total heterogeneity): 0.2291 (SE = 0.4014)
## tau (square root of estimated tau^2 value): 0.4786
## I^2 (total heterogeneity / total variability): 40.41%
## H^2 (total variability / sampling variability): 1.68
##
## Test for Heterogeneity:
## Q(df = 4) = 6.7120, p-val = 0.1519
##
## Model Results:
##
## estimate se zval pval ci.lb ci.ub
## -0.0201 0.3371 -0.0597 0.9524 -0.6808 0.6406
##
## ---
## Signif. codes: 0 '***' 0.001 '**' 0.01 '*' 0.05 '.' 0.1 ' ' 1

## all effect sizes (violates important assumptions!)
mafulldata <- rma(lgor, lgorv, method="DL", data=fulldata)
trimfill(mafulldata)

##
## Estimated number of missing studies on the right side: 31 (SE = 7.7540)
##
## Random-Effects Model (k = 171; tau^2 estimator: DL)
##
## tau^2 (estimated amount of total heterogeneity): 0.0185 (SE = 0.0101)
## tau (square root of estimated tau^2 value): 0.1359
## I^2 (total heterogeneity / total variability): 20.20%
## H^2 (total variability / sampling variability): 1.25
##
## Test for Heterogeneity:
## Q(df = 170) = 213.0417, p-val = 0.0140
##
## Model Results:
##
## estimate se zval pval ci.lb ci.ub
## 0.1121 0.0245 4.5682 <.0001 0.0640 0.1601 ***
##
## ---
## Signif. codes: 0 '***' 0.001 '**' 0.01 '*' 0.05 '.' 0.1 ' ' 1

funnel(mafulldata)


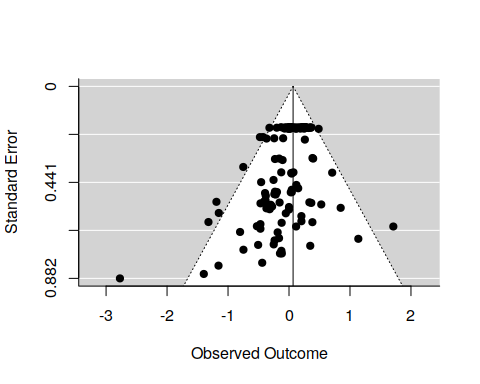


png('funnel2.png', width=800,height=450)
funnel(mafulldata)
dev.off()

## png
## 2

## Egger's test
regtest(rma(lgor, lgorv, method="DL", data=official))

##
## Regression Test for Funnel Plot Asymmetry
##
## model: mixed-effects meta-regression model
## predictor: standard error
##
## test for funnel plot asymmetry: z = -1.6671, p = 0.0955

### Sensitivity Analyses

A total of 140 effect sizes were calculated across all studies (however, 87 of these were from a single study representing three treatment-comparison constrasts: Dunford, 2000). All other studies had between 1 and 19 effect sizes. However, to ensure that the above analyses are not biased as a result of the particular effect sizes selected for analysis, we ran sensitivity analyses using all effect sizes. To deal with the dependencies for multiple effect sizes from a single study, we used the robust standard errors method, clustering on studyid+txid. We also ran the analyses clustering simply on studyid as a more robust check.

## number of positive, negative and 0 effect sizes
fulldata$esdirect <- factor(fulldata$esdirect, labels = c("Favors tx","Favors cg", "Equal"))
table(fulldata$esdirect)

##
## Favors tx Favors cg Equal
## 66 71 3

## number of effect sizes per studyid+txid
fulldata$studytxid <- as.numeric(paste(es$studyid,es$txid,sep="."))
as.data.frame(table(fulldata$studytxid))

## Var1 Freq
## 1 1.1 53
## 2 1.2 17
## 3 1.3 17
## 4 2.1 4
## 5 2.2 4
## 6 4.1 1
## 7 5.1 19
## 8 12.1 1
## 9 27.1 2
## 10 30.1 6
## 11 45.1 4
## 12 101.1 4
## 13 105.1 2
## 14 157.1 6

fulldata$studyid <- as.numeric(fulldata$studyid)
fulldata$published <- ifelse(fulldata$pubtype==3,1,0)
fulldata$random <- ifelse(fulldata$txrandom<4,1,0)
fulldata$official <- ifelse(fulldata$dvsource==1,1,0)
fulldata$victim <- ifelse(fulldata$dvsource!=1 & fulldata$dvsource!=3,1,0)
## run the models
fullm1 <- rma.mv(lgor, lgorv, random = list(~1 | studytxid), data=fulldata )
fullm2 <- rma.mv(lgor ~ random+published+official+victim,
 lgorv, random = list(~1 | studytxid), data=fulldata )
fullm3 <- rma.mv(lgor, lgorv, random = list(~1 | studyid), data=fulldata )
fullm4 <- rma.mv(lgor ~ random+published+official+victim,
 lgorv, random = list(~1 | studyid), data=fulldata )
fullm5 <- rma.mv(lgor ~ official+victim,
 lgorv, random = list(~1 | studyid), data=fulldata )

print(fullm1)

##
## Multivariate Meta-Analysis Model (k = 140; method: REML)
##
## Variance Components:
##
## estim sqrt nlvls fixed factor
## sigma^2 0.1468 0.3831 14 no studytxid
##
## Test for Heterogeneity:
## Q(df = 139) = 131.9583, p-val = 0.6516
##
## Model Results:
##
## estimate se zval pval ci.lb ci.ub
## -0.1420 0.1243 -1.1419 0.2535 -0.3856 0.1017
##
## ---
## Signif. codes: 0 '***' 0.001 '**' 0.01 '*' 0.05 '.' 0.1 ' ' 1

print(fullm2)

##
## Multivariate Meta-Analysis Model (k = 140; method: REML)
##
## Variance Components:
##
## estim sqrt nlvls fixed factor
## sigma^2 0.1920 0.4382 14 no studytxid
##
## Test for Residual Heterogeneity:
## QE(df = 135) = 117.8320, p-val = 0.8535
##
## Test of Moderators (coefficients 2:5):
## QM(df = 4) = 1.8128, p-val = 0.7701
##
## Model Results:
##
## estimate se zval pval ci.lb ci.ub
## intrcpt -0.0301 0.3594 -0.0838 0.9332 -0.7345 0.6743
## random 0.0813 0.3083 0.2636 0.7921 -0.5230 0.6855
## published -0.2297 0.2999 -0.7661 0.4436 -0.8175 0.3580
## official -0.1058 0.1713 -0.6178 0.5367 -0.4415 0.2299
## victim 0.0252 0.0442 0.5698 0.5688 -0.0615 0.1118
##
## ---
## Signif. codes: 0 '***' 0.001 '**' 0.01 '*' 0.05 '.' 0.1 ' ' 1

print(fullm3)

##
## Multivariate Meta-Analysis Model (k = 140; method: REML)
##
## Variance Components:
##
## estim sqrt nlvls fixed factor
## sigma^2 0.2240 0.4733 11 no studyid
##
## Test for Heterogeneity:
## Q(df = 139) = 131.9583, p-val = 0.6516
##
## Model Results:
##
## estimate se zval pval ci.lb ci.ub
## -0.2100 0.1694 -1.2395 0.2152 -0.5421 0.1221
##
## ---
## Signif. codes: 0 '***' 0.001 '**' 0.01 '*' 0.05 '.' 0.1 ' ' 1

print(fullm4)

##
## Multivariate Meta-Analysis Model (k = 140; method: REML)
##
## Variance Components:
##
## estim sqrt nlvls fixed factor
## sigma^2 0.3017 0.5493 11 no studyid
##
## Test for Residual Heterogeneity:
## QE(df = 135) = 117.8320, p-val = 0.8535
##
## Test of Moderators (coefficients 2:5):
## QM(df = 4) = 2.4998, p-val = 0.6447
##
## Model Results:
##
## estimate se zval pval ci.lb ci.ub
## intrcpt 0.2060 0.4620 0.4460 0.6556 -0.6994 1.1114
## random -0.1532 0.4411 -0.3472 0.7284 -1.0177 0.7114
## published -0.5892 0.4432 -1.3295 0.1837 -1.4579 0.2794
## official -0.0605 0.1779 -0.3403 0.7336 -0.4092 0.2881
## victim 0.0259 0.0442 0.5854 0.5583 -0.0608 0.1125
##
## ---
## Signif. codes: 0 '***' 0.001 '**' 0.01 '*' 0.05 '.' 0.1 ' ' 1

print(fullm5)

##
## Multivariate Meta-Analysis Model (k = 140; method: REML)
##
## Variance Components:
##
## estim sqrt nlvls fixed factor
## sigma^2 0.2269 0.4764 11 no studyid
##
## Test for Residual Heterogeneity:
## QE(df = 137) = 121.4030, p-val = 0.8264
##
## Test of Moderators (coefficients 2:3):
## QM(df = 2) = 0.6436, p-val = 0.7248
##
## Model Results:
##
## estimate se zval pval ci.lb ci.ub
## intrcpt -0.1800 0.1960 -0.9184 0.3584 -0.5640 0.2041
## official -0.0743 0.1733 -0.4287 0.6681 -0.4139 0.2653
## victim 0.0262 0.0442 0.5921 0.5538 -0.0605 0.1128
##
## ---
## Signif. codes: 0 '***' 0.001 '**' 0.01 '*' 0.05 '.' 0.1 ' ' 1

## using robumeta
fullm6 <- robu(formula = lgor ~ random, var.eff.size = lgorv, data = fulldata,
 studynum = studyid, small = TRUE)
fullm6

## RVE: Correlated Effects Model with Small-Sample Corrections
##
## Model: lgor ~ random
##
## Number of studies = 11
## Number of outcomes = 140 (min = 1 , mean = 12.7 , median = 4 , max = 87 )
## Rho = 0.8
## I.sq = 50.49487
## Tau.sq = 0.3056051
##
## Estimate StdErr t-value dfs P(|t|>) 95% CI.L 95% CI.U Sig
## 1 X.Intercept. -0.412 0.360 -1.15 4.74 0.307 -1.353 0.529
## 2 random 0.220 0.408 0.54 8.11 0.604 -0.718 1.158
## ---
## Signif. codes: < .01 *** < .05 ** < .10 *
## ---
## Note: If df < 4, do not trust the results
